# Cost-effectiveness of janus kinase inhibitors for rheumatoid arthritis: A systematic review and meta-analysis of cost-utility studies

**DOI:** 10.3389/fphar.2022.1090361

**Published:** 2022-12-13

**Authors:** S. Sajith Kumar, Madhumitha Haridoss, Krishnamurthy Venkataraman, Bhavani Shankara Bagepally

**Affiliations:** ^1^ Health Technology Assessment Resource Centre, ICMR-National Institute of Epidemiology, Chennai, India; ^2^ Chennai Meenakshi Multispecialty Hospital, Chennai, India

**Keywords:** rheumatoid arthritis, janus kinase inhibitors, cost-effectiveness, QALY, evidence syntheses

## Abstract

**Introduction:** Janus kinase inhibitors (JAK-i), a class of targeted synthetic disease-modifying antirheumatic drugs (tDMARDs), are suggested as second or third-line therapies in rheumatoid arthritis (RA). Synthesized cost-effective evidence would aid in informed decision-making given the similar clinical effectiveness of JAKi, but incongruent cost-effectiveness reports.

**Methods:** Literature search was conducted in PubMed, Embase, Scopus, and Tufts Medical Centers’ cost-effective analysis registry. We pooled the incremental net benefit (INB) with 95% confidence interval (CI) using random-effects model and the heterogeneity was assessed using Cochrane-Q test and I2 statistic. Modified economic evaluation bias checklist was used to assess the quality of selected studies. Publication bias was assessed using a funnel plot and Egger’s test. The Grading of Recommendation, Assessment, Development, and Evaluation (GRADE) assessment was performed to assess the certainty of outcomes presented.

**Results:** We included seventeen relevant studies for systematic review, of which fifteen were eligible for meta-analysis. The meta-analysis results showed that JAK-i is cost-effective compared to csDMARDS/bDMARDs with a pooled INB (INBp) of $19,886 (95% CI, 1,635 to 38,137) but with considerable heterogeneity (I2 = 99.14). As a second-line treatment for csDMARD failed RA, JAK-i is cost-effective than csDMARD/bDMARD with a pooled INB of $23,144 (74.1–46,214) and high heterogeneity (I2 = 99.67). But on a separate analysis JAK-i as second-line treatment is not cost-effective than TNF-a-i (INBp = $25,813, -5,714 to 57,340). However, leave-one-out analysis found that omitting a single outlier makes JAK-i cost-effective. Further, JAK-i is not cost-effective as a third-line treatment for csDMARD-TNF-a-I failed RA, compared to csDMARDs/bDMARDs with INBp $26,157 (-7,284 to 59,598).

**Conclusion:** Meta-analysis suggests that JAK-i is cost-effective when used after csDMARD failure but not cost-effective when used after csDMARD-TNF-a-i failure with low certainty of evidence.

**Clinical Trial Registration:** https://www.crd.york.ac.uk/prospero/display_record.php?ID=CRD42021222541, identifier CRD42021222541

## Introduction

Rheumatoid arthritis (RA), a chronic autoimmune condition that affects the synovial joints causing pain and inflammation, can significantly reduce a person’s quality of life when left untreated. (Smolen et al., 2016) Early diagnosis and treatment may prevent permanent joint damage and functional disability, particularly in patients with active disease. (Smolen et al., 2016) According to current treatment guidelines, methotrexate (MTX), a conventional synthetic disease-modifying antirheumatic drug (csDMARD) is used as the first-line treatment for RA with or without low doses of corticosteroids. (Smolen et al., 2020) However, in patients who are not suitable to be treated with MTX, such as comorbidities or contraindications (such as hepatitis-B virus infection) or adverse events (AEs), other csDMARDs such as hydroxychloroquine, leflunomide and sulfasalazine are recommended. (Smolen et al., 2020) About 20%–30% of RA patients are resistant to multiple DMARDs. (Smolen et al., 2020) Therefore, in patients who have failed to respond to previous csDMARDs, targeted synthetic DMARDs such as Janus kinase inhibitors (JAK-i) and biological disease-modifying antirheumatic drugs (bDMARDs) including Tumor necrosis factor-alpha inhibitors (TNF-a-i), Interleukin-6 inhibitors (IL-6-i), B-cell inhibitors are currently considered second-line treatments. However, current guidelines do not recommend any specific drug for RA patients once csDMARD treatment has failed. (Lau et al., 20182019).

Given that JAK-i [Tofacitinib (TOFA), Baricitinib (BARI), Upadacitinib (UPA), Filgotinib (FILG)] are as clinically effective as bDMARDs (Strand et al., 2016; Fleischmann et al., 2017; Taylor et al., 2017; Grimm et al., 2021), clinicians and policymakers would consider the cost-effectiveness of these drugs when determining the treatment for RA patients. (Russell et al., 1996) Cost-effectiveness analyses (CEA) collate evidence from multiple sources to comparatively analyse considering both the costs and benefits of the treatment. (Russell et al., 1996) Therefore, CEAs have been regarded as the “gold standard” for creating fair estimates of the value of health interventions to guide decision-making. (Siegel et al., 1996) While many studies have reported on the cost-effectiveness of JAK-i in RA treatment, there is currently no systematic review of such economic evaluations. Therefore, a comprehensive systematic evaluation and analysis of existing cost-effectiveness evidence are required. Hence, we conducted a systematic review of the available evidence on the cost-effectiveness of JAK-i for RA treatment and calculated the pooled incremental net benefit (INB).

## Methods

The study protocol was conducted according to the Preferred Reporting Items for Systematic Reviews and Meta-analyses (PRISMA) (Moher et al., 2015). This work is a part of an SRMA protocol that has been registered with PROSPERO under CRD 42021222541.

### Data sources and eligibility criteria

The initial literature search was conducted on 12th February 2021 in PubMed, Embase, Scopus, and the Tufts Medical Centers’ cost-effective analysis registry (Registry). The search terms are constructed based on the domains of the PICO approach (Population, Intervention, Comparator, Outcome). Published cost-utility studies (CUA) of adult subjects with moderate to severe RA treated with JAK-i alone or in combinations of other DMARDs were included in the study. Incremental cost-effectiveness ratios (ICER) per quality-adjusted life years (QALYs) or incremental net benefit (INB) was the outcome measure. Studies with effectiveness measured other than in QALYs, abstracts, grey literature, and methodological articles were excluded. We conducted an updated search on 5th May 2022 using the same search strategy and inclusion criteria. The detailed search strategy is reported in Appendix 1.

### Screening and selection of studies

**FIGURE 1:**
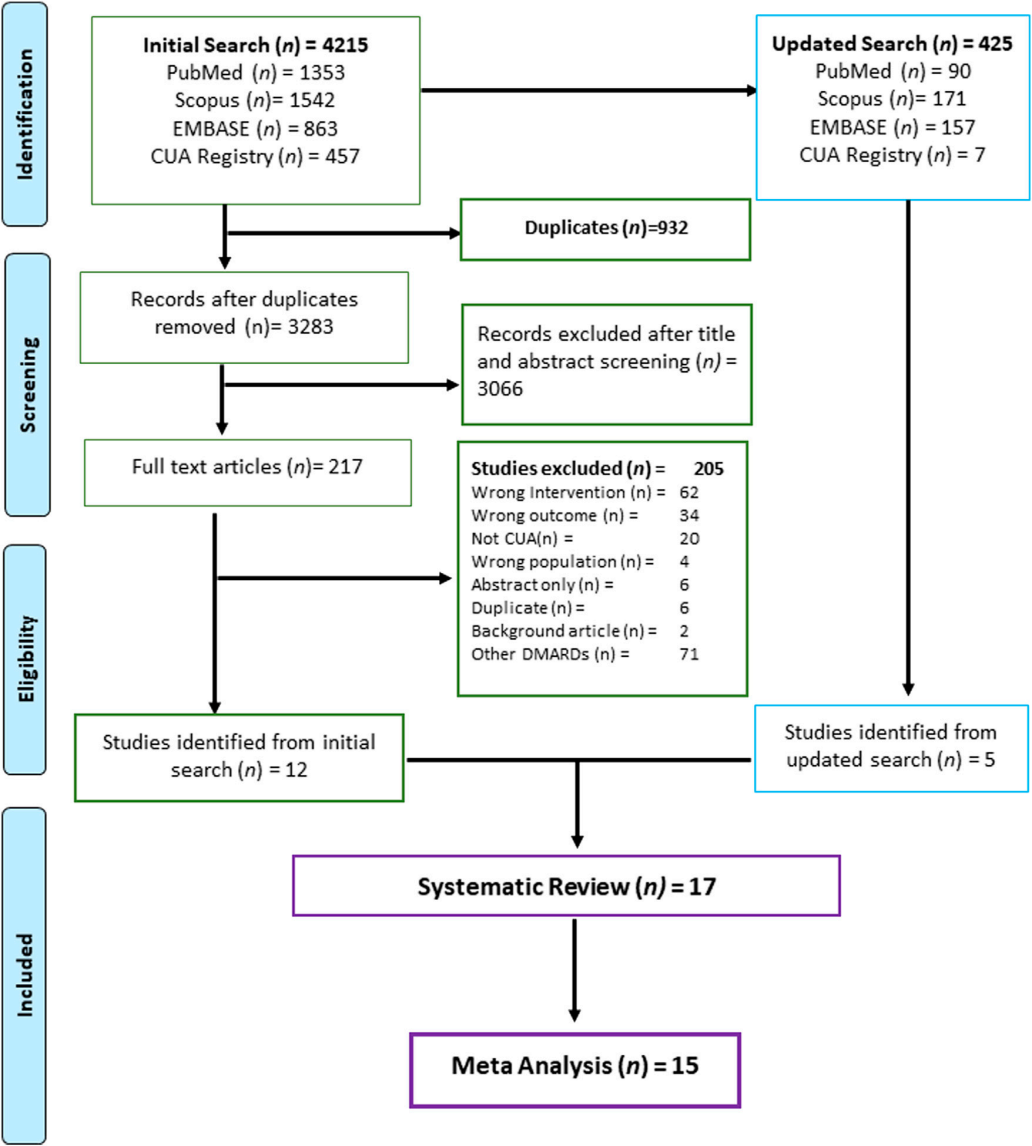
List of studies identified and included for systematic review and meta analysis.

We identified 4,215 studies from the initial search and 425 studies from the updated search. All studies that met the eligibility criteria were screened independently for titles and abstracts by two independent reviewers (BSB and SK) using the Rayyan-web application (Ouzzani et al., 2016). Reviewers (BSB and SK) independently reviewed the full-text studies, and based on independent assessors’ mutual agreement, the list of studies (*n* = 17) meeting inclusion criteria was finalized. The detailed screening process is appended in Figure 1.

### Data extraction, cleaning and analysis

Two reviewers (BSB and SK) independently extracted and verified data from the identified studies (*n* = 17). A pre-structured data extraction form was used to extract the details of the study; author, year, country, target population, intervention, comparator, and the model characteristics, including model type, study perspective, time horizons, discount rate, and currency year. Economic parameters such as costs (C), clinical effectiveness in terms of QALY (E), incremental values (ΔC and ΔE), ICERs, INBs, and their measures of dispersion, willingness to pay (WTP), and threshold (K) were extracted. WebPlotDigitaliser (Rohatgi, 2021) was used to extract data from cost-effective (CE) plane graphs. For studies without a CE plane graph, covariance was estimated using one-thousand Monte-Carlo simulations from the extracted costs and effectiveness (Appendix 2).

For the meta-analysis, outcome of interest was pooled INB, defined as K*ΔE-ΔC, where K was the WTP threshold, ΔC and ΔE are the incremental cost and incremental effectiveness (i.e., the difference in cost/effectiveness between intervention and comparator) respectively. A positive INB value favours intervention, i.e., intervention is cost-effective, whereas a negative INB favours the comparator, i.e., intervention is not cost-effective. INB is used as an effect measure over ICER because of the statistical advantages of INB and uncertainties in interpreting ICER values. (O’Mahony et al., 2015; Bagepally et al., 2019; Paulden, 2020).

We followed the data preparation method and analysis reported and used elsewhere. (Bagepally et al., 2022) We have calculated the pooled INB and its variances for each intervention comparator duo, following the CUA methodology detailed in Bagepally et al. (2019); Bagepally et al. (2020). Inflation adjustment and currency conversion to the US dollar was made using the consumer price index (CPI) and purchasing power parities (PPP) for the year 2021 (Appendix 2). (World Economic Outlook Database, 2021).

Meta-analysis was applied to pool the INBs using a random-effects model based on the DerSimonian and Laird methods. I^2^ statistics, leave-one-out analysis and Galbraith plot were used to assess sensitivity and heterogeneity. The source of heterogeneity was also explored using sub-group analysis, and subgroup-specific pooled INBs were reported in the results. Furthermore, publication bias was assessed using funnel plots and Eggers’ test. All data were prepared using Microsoft Excel version 2019 (Corporation. M, 2018) and analyzed using Stata software version 17 (StataCorp, 2019).

### Risk of bias assessment and quality assessment

The modified economic evaluation bias (ECOBIAS) checklist was used to evaluate the reporting quality and bias of the identified studies (Adarkwah et al., 2016). ECOBIAS assesses each study’s overall biases, model-specific biases and internal consistency. Furthermore, GRADE (Grading of Recommendation, Assessment, Development, and Evaluation) was used to assess the quality of evidence (Guyatt et al., 2011; Hultcrantz et al., 2017). The evidence was graded for the cost-effectiveness of JAK-i compared to other DMARDS. The GRADE assessment use risk of bias, inconsistency, indirectness, imprecision, publication bias, and other considerations to classify the quality of the evidence as high, moderate, low, or very low (Guyatt et al., 2011; Hultcrantz et al., 2017).

## Results

### Characteristics of included studies

We included seventeen (Lee et al., 2015; Jansen et al., 2017; Claxton et al., 2018; Chen et al., 2019; Fournier et al., 2019; Muszbek et al., 2019; Schlueter et al., 2019; Navarro et al., 2020; Tian et al., 2020; Van De Laar et al., 2020; Li et al., 2021a; Li et al., 2021b; Fatemi et al., 2021; Ha et al., 2021; Tan et al., 2021; Kuwana et al., 2022; Tan et al., 2022) relevant studies for systematic review, of which fifteen studies (Lee et al., 2015; Jansen et al., 2017; Claxton et al., 2018; Chen et al., 2019; Muszbek et al., 2019; Schlueter et al., 2019; Navarro et al., 2020; Tian et al., 2020; Van De Laar et al., 2020; Li et al., 2021a; Fatemi et al., 2021; Ha et al., 2021; Tan et al., 2021; Kuwana et al., 2022; Tan et al., 2022) were eligible for meta-analysis (Figure 1). All the studies with JAK-i as an intervention were included for the meta-analysis (*n* = 15), whereas studies that compared JAK-i *versus* JAK-i (*n* = 2) were included for systematic review only (Fournier et al., 2019; Li et al., 2021b). The characteristics of the included studies in the systematic review and meta-analysis are summarised in Table 1.

**TABLE 1:** General characteristics of the studies included in systematic review and meta-analysis.

Author_ year	Country	Setting	Perspective	Target population	Time horizon	Discount rate for costs (%)	References year	Intervention	Comparator	Remarks
Van De Laar et al. (2020)	Netherland	Country	Societal	Severe RA	5 Year	4.0	2019	csDMARD—Ada Seq	csDMARDs—Bari Seq	Dominated
Chen et al. (2019)	Taiwan	Country	Payer	Moderate to severe RA	Lifetime	3.0	2015	Tofa + MTX	Ada + MTX	Cost effective
Claxton et al. (2018)	United States of America	Risk Group	Health System	Moderate to severe RA	Lifetime	3.0	2015	MTX—Tofa—Ada—Aba—Toci—Ritu	MTX—Eta—Ada—Aba—Toci—Ritu	Cost saving
Fatemi et al. (2021)	Iran	Risk Group	Payer	Severe RA	Lifetime	7.2	2019	Tofa + MTX	Eta-Ada-Ritu	Cost-effective
Fournier et al. (2019) *	United States	Country	Health System	Moderate to severe RA	10 Year	3.0	2018	Sari—Tofa—csDMARD	Ada—Tofa—csDMARD	Dominant
Jansen et al. (2017)	United States	Country	Societal	Moderate to severe RA	Lifetime	3.0	2016	Eta—Ada—Aba—Toci- Tofa—Ritu—csDMARD	csDMARD	Cost effective
Kuwana et al. (2022)	Japan	Country	Health System	Moderate to severe RA	Lifetime	3.0	2020	Bari + MTX	csDMARD	Cost effective
Tian et al. (2020)	China	Country	Health System	Moderate to severe RA	Lifetime	5.0	2018	Tofa-Tnfi-Toci-PC	Toci + PC	Cost saving
Schlueter et al. (2019)	Spain	Country	Health System	Moderate to severe RA	Lifetime	3.0	2018	Bari	Ada	Cost-effective
Lee et al. (2015)	South Korea	Country	Societal	Moderate to severe RA	Lifetime	5.0	2013	Tofa + MTX—Ada + MTX + Eta + MTX—csDMARD	Ada + MTX + Eta + MTX–csDMARD	Cost effective
Muszbek et al. (2019)	United States	Country	Health System	Moderate to severe RA	Lifetime	3.0	2017	Tofa + MTX	Sari + MTX	Dominant
Navarro et al. (2020)	Spain	Country	Health System	Moderate to severe RA	Lifetime	3.0	2018	Tofa + MTX—Toci + MTX—Aba + MTX- Ritu + MTX	Toci + MTX—Abat + MTX—ritu + MTX—certo + MTX	Dominant
Smolen et al. (2016), Li et al. (2021a)	China	Risk Group	Health System	Moderate to severe RA	Lifetime	3.0	2019	Bari-Ada-Eta-Toci-PC	Ada + MTX	Cost-effective
Smolen et al. (2020), Li et al. (2021b) *	China	Risk Group	Health System	Moderate to severe RA	Lifetime	3.0	2019	TT - Ritu—Tofa	Eta—Aba—Tofa	Cost effective
Ha et al. (2021)	South Korea	Risk Group	Societal	Moderate RA	Lifetime	5.0	2019	Tofa—BDMARDs	csDMARDs	Cost effective
Smolen et al. (2016), Tan et al. (2021)	China	Country	Health System	Moderate to severe RA	Lifetime	3.0	2019	Tofa—Eta—Ritu - Toci	Eta—Ritu—Toci	Dominant
Smolen et al. (2020), Tan et al. (2022)	China	Risk Group	Health System	Moderate to severe RA	Lifetime	3.0	2019	Eta—Tofa—Ritu—Toci	MTX	Not cost-effective

* Systematic review, HIC, High-income country; UMIC, Upper middle-income country; LMIC, Lower middle-income country; NR, not, reported; RA, rheumatoid arthritis; MTX, methotrexate; Aba, abatacept; Ritu, Rituximab; Ada, Adalimumab; Toci, Tocilizumab; Goli, Golimumab; Eta, Etanercept; TT, tripple therapy; Tofa, Tofacitinib; Bari, Baricitinib; Certo, Certolizumab; Sari, Sarilumumab; Lefl, Leflunomide; csdmards, conventional synthetic disease-modifying anti rheumatic drugs; Seq, Sequential; PC, Palliative care.

Thirteen studies (Lee et al., 2015; Claxton et al., 2018; Chen et al., 2019; Muszbek et al., 2019; Schlueter et al., 2019; Navarro et al., 2020; Tian et al., 2020; Van De Laar et al., 2020; Li et al., 2021a; Fatemi et al., 2021; Ha et al., 2021; Tan et al., 2021; Kuwana et al., 2022) assessed the cost-effectiveness of JAK-i as second line treatment in RA patients who showed an inadequate response to csDMARDs. Five studies (Jansen et al., 2017; Claxton et al., 2018; Navarro et al., 2020; Tan et al., 2021; Tan et al., 2022) assessed the cost-effectiveness of JAK-i compared to csDMARD/bDMARDs as the third-line treatment for RA patients who showed an inadequate response to TNF-a-i following csDMARD failure. There are no studies which assessed the cost-effectiveness of JAK-i as first line treatment in early RA patients.

Eleven studies (Lee et al., 2015; Jansen et al., 2017; Claxton et al., 2018; Chen et al., 2019; Fournier et al., 2019; Muszbek et al., 2019; Schlueter et al., 2019; Navarro et al., 2020; Van De Laar et al., 2020; Ha et al., 2021; Kuwana et al., 2022) were from High-income countries (HIC), five studies from upper-middle-income countries (UMICs) (Tian et al., 2020; Li et al., 2021a; Li et al., 2021b; Tan et al., 2021; Tan et al., 2022) and only one study from lower middle-income country (LMICs). (Fatemi et al., 2021) ICER was calculated from a Health system perspective in eleven studies, (Claxton et al., 2018; Fournier et al., 2019; Muszbek et al., 2019; Schlueter et al., 2019; Navarro et al., 2020; Tian et al., 2020; Li et al., 2021a; Li et al., 2021b; Tan et al., 2021; Kuwana et al., 2022; Tan et al., 2022) societal perspective in four studies, (Lee et al., 2015; Jansen et al., 2017; Van De Laar et al., 2020; Ha et al., 2021) and payer’s perspective in two studies. (Chen et al., 2019; Fatemi et al., 2021) All studies used a model-based analytical approach, out of which eleven studies (Lee et al., 2015; Jansen et al., 2017; Claxton et al., 2018; Chen et al., 2019; Schlueter et al., 2019; Navarro et al., 2020; Li et al., 2021a; Li et al., 2021b; Tan et al., 2021; Kuwana et al., 2022; Tan et al., 2022) used an event simulation model, and six studies (Fournier et al., 2019; Muszbek et al., 2019; Tian et al., 2020; Van De Laar et al., 2020; Fatemi et al., 2021; Ha et al., 2021) used a Markov model. All the studies except Fournier et al. (2019) (ten-year horizon) and Van De Laar et al. (2020) (five-year horizon) used a lifetime horizon for the calculation of costs and QALY (*n* = 15). (Lee et al., 2015; Jansen et al., 2017; Claxton et al., 2018; Chen et al., 2019; Muszbek et al., 2019; Schlueter et al., 2019; Navarro et al., 2020; Tian et al., 2020; Li et al., 2021a; Li et al., 2021b; Fatemi et al., 2021; Ha et al., 2021; Tan et al., 2021; Kuwana et al., 2022; Tan et al., 2022).

Most studies (*n* = 12) (Jansen et al., 2017; Claxton et al., 2018; Chen et al., 2019; Fournier et al., 2019; Muszbek et al., 2019; Schlueter et al., 2019; Navarro et al., 2020; Li et al., 2021a; Li et al., 2021b; Tan et al., 2021; Kuwana et al., 2022; Tan et al., 2022) used a 3 per cent discount rate for costs, three studies used a 5 percent discount rate, (Lee et al., 2015; Tian et al., 2020; Ha et al., 2021) Van De Laar et al. (2020) used 4 percent and Fatemi et al. (2021) used 7.2 percent per annum rate for discounting costs. Country-specific willingness to pay threshold (Jansen et al., 2017; Claxton et al., 2018; Chen et al., 2019; Fournier et al., 2019; Muszbek et al., 2019; Schlueter et al., 2019; Navarro et al., 2020; Van De Laar et al., 2020; Ha et al., 2021; Kuwana et al., 2022) was used in ten studies whereas GDP-based WTP (Fatemi et al., 2021), (Lee et al., 2015), (Li et al., 2021a), (Tian et al., 2020; Tan et al., 2021; Tan et al., 2022), (Tian et al., 2020; Tan et al., 2021; Tan et al., 2022) was used in seven studies.

Studies are classified into five scenarios based on the reported outcome and dispersion measures (Bagepally et al., 2022). Most of the studies were in scenario five (*n* = 11) (Lee et al., 2015; Claxton et al., 2018; Fournier et al., 2019; Muszbek et al., 2019; Navarro et al., 2020; Li et al., 2021a; Li et al., 2021b; Ha et al., 2021; Tan et al., 2021; Kuwana et al., 2022; Tan et al., 2022), followed by four studies in scenario four (Chen et al., 2019; Schlueter et al., 2019; Tian et al., 2020; Fatemi et al., 2021) and one study each under scenario one (Jansen et al., 2017) and three (Van De Laar et al., 2020). INB variance of Schlueter et al. (2019) was used for five other studies (Claxton et al., 2018; Fournier et al., 2019; Navarro et al., 2020; Li et al., 2021a; Ha et al., 2021; Kuwana et al., 2022), Tian et al. (2020) for three studies (Lee et al., 2015; Tan et al., 2021; Tan et al., 2022) and Fatemi et al. (2021) for two studies (Fournier et al., 2019; Muszbek et al., 2019).

### Risk of bias assessment

Nearly 94 per cent of the studies justified the perspective used for analysis, indicating a narrow perspective bias. Similarly, most the studies used the adequate comparator for analysis; hence the treatment comparator bias was low. Reporting and dissemination bias is 52 per cent, whereas limited time horizon bias is low since 94 per cent of the studies justified the time horizons. The methods of data identification were transparent for 59 per cent of studies. Limited scope bias is very high (65 per cent); also, internal consistency was not appropriately evaluated (Supplementary Figure S1).

### Cost-effectiveness of JAK-i compared to csDMARDs/bDMARDs

The meta-analysis includes studies that evaluated the cost-effectiveness of JAK-i against csDMARDs/bDMARDs for RA patients with csDMARD failure or csDMARD-TNF-a-i failure. (Lee et al., 2015; Jansen et al., 2017; Claxton et al., 2018; Chen et al., 2019; Muszbek et al., 2019; Schlueter et al., 2019; Navarro et al., 2020; Tian et al., 2020; Van De Laar et al., 2020; Li et al., 2021a; Fatemi et al., 2021; Ha et al., 2021; Tan et al., 2021; Kuwana et al., 2022; Tan et al., 2022). The pooled INB (INBp) was $19,886 and 95% CI (1,635 to 38,137) which shows JAK-i is significantly cost-effective compared to csDMARDs and bDMARDS, however with a considerable heterogeneity (I^2^ = 99.14) (Supplementary Figure S2). As per the leave-one-out sensitivity analysis, two individual studies significantly influence the overall estimate (Claxton et al., 2018; Tan et al., 2022). Leaving Claxton et al., cause a decrease in INBp values ($13,512 and 95% CI = 3,317 to 23,707) and Tan et al., cause an increase in INBp ($25,720 and 95% CI = 7,043 to 44,398) (Supplementary Figure S3). The Galbraith plot shows all the studies except two within the 95 per cent confidence interval indicating the possibility of low inconsistency across studies (Supplementary Figure S4). The funnel plot showed asymmetry (Supplementary Figure S5); however, the Egger’s test with a higher *p*-value (*p* = 0.561) indicates no significant variability among the studies and no publication bias.

### Subgroup analysis

Sub-group and sensitivity analyses were performed to explore the source of heterogeneity. Subgroup analysis based on study perspectives showed that JAK-i is cost-effective only from a societal perspective (*n* = 4) (Lee et al., 2015; Jansen et al., 2017; Van De Laar et al., 2020; Ha et al., 2021) with a INBp of $9,976 (6,596 to 13,355) and no heterogeneity (I^2^ = 0). However, the intervention is not cost-effective neither from a health-system perspective (*n* = 9) (Claxton et al., 2018; Muszbek et al., 2019; Schlueter et al., 2019; Navarro et al., 2020; Tian et al., 2020; Li et al., 2021a; Tan et al., 2021; Kuwana et al., 2022; Tan et al., 2022) (INBp = $20,681, -2,965 to 44,328) nor from a payers perspective (*n* = 2) (Chen et al., 2019; Fatemi et al., 2021) (INBp = 14,456, -71,483 to 100,395) with a high heterogeneity in health-system perspective subgroup (Supplementary Figure S6).

Subgroup analysis based on income-classification found that JAK-i is cost-effective in HICs (*n* = 10) (Lee et al., 2015; Jansen et al., 2017; Claxton et al., 2018; Chen et al., 2019; Muszbek et al., 2019; Schlueter et al., 2019; Navarro et al., 2020; Van De Laar et al., 2020; Ha et al., 2021; Kuwana et al., 2022) with INBp $31,502 (6,440 to 56,564) and high heterogeneity (I^2^ = 99.35). However, the results were not significant for UMICs (*n* = 4) (Tian et al., 2020; Li et al., 2021a; Tan et al., 2021; Tan et al., 2022) with a pooled INB of -$791 (-25,230 to 23,648) with substantial heterogeneity (I^2^ = 81.66) (Supplementary Figure S7).

The median threshold used for the analysis is $41,118. JAK-i is significantly cost-effective for the studies when threshold is more than median value (*n* = 8) (Jansen et al., 2017; Claxton et al., 2018; Chen et al., 2019; Muszbek et al., 2019; Schlueter et al., 2019; Navarro et al., 2020; Van De Laar et al., 2020; Kuwana et al., 2022) with INBp $38,972 (95% CI 5,289 to 72,655) and high heterogeneity (I^2^ = 99.47). However, JAK-i is not cost-effective for studies when the threshold is less than the median (*n* = 7) (Lee et al., 2015; Tian et al., 2020; Li et al., 2021a; Fatemi et al., 2021; Ha et al., 2021; Tan et al., 2021; Tan et al., 2022) with an INBp of 7,455 (-1,074 to 15,984) (Supplementary Figure S8).

On subgroup analysis based on scenario, JAK-i is not cost-effective in scenario four (*n* = 4) (Chen et al., 2019; Schlueter et al., 2019; Tian et al., 2020; Fatemi et al., 2021) (INBp = $11,060, -1,345 to 23,464) or scenario five (*n* = 9) (Lee et al., 2015; Claxton et al., 2018; Muszbek et al., 2019; Navarro et al., 2020; Li et al., 2021a; Ha et al., 2021; Tan et al., 2021; Kuwana et al., 2022; Tan et al., 2022) (INBp = $19,145, -5,374 to 44,264) (Supplementary Figure S9).

Similarly, on subgroup analysis based on time horizon (*n* = 14), JAK-i is cost-effective with an INBp of $20,281 (1,855 to 38,707) though with high heterogeneity (I^2^ = 99.2%) (Supplementary Figure S10).

### Separate analysis for the cost-effectiveness of JAK-i *versus* csDMARD/bDMARD as second-line treatment for csDMARD failed RA

**FIGURE 2:**
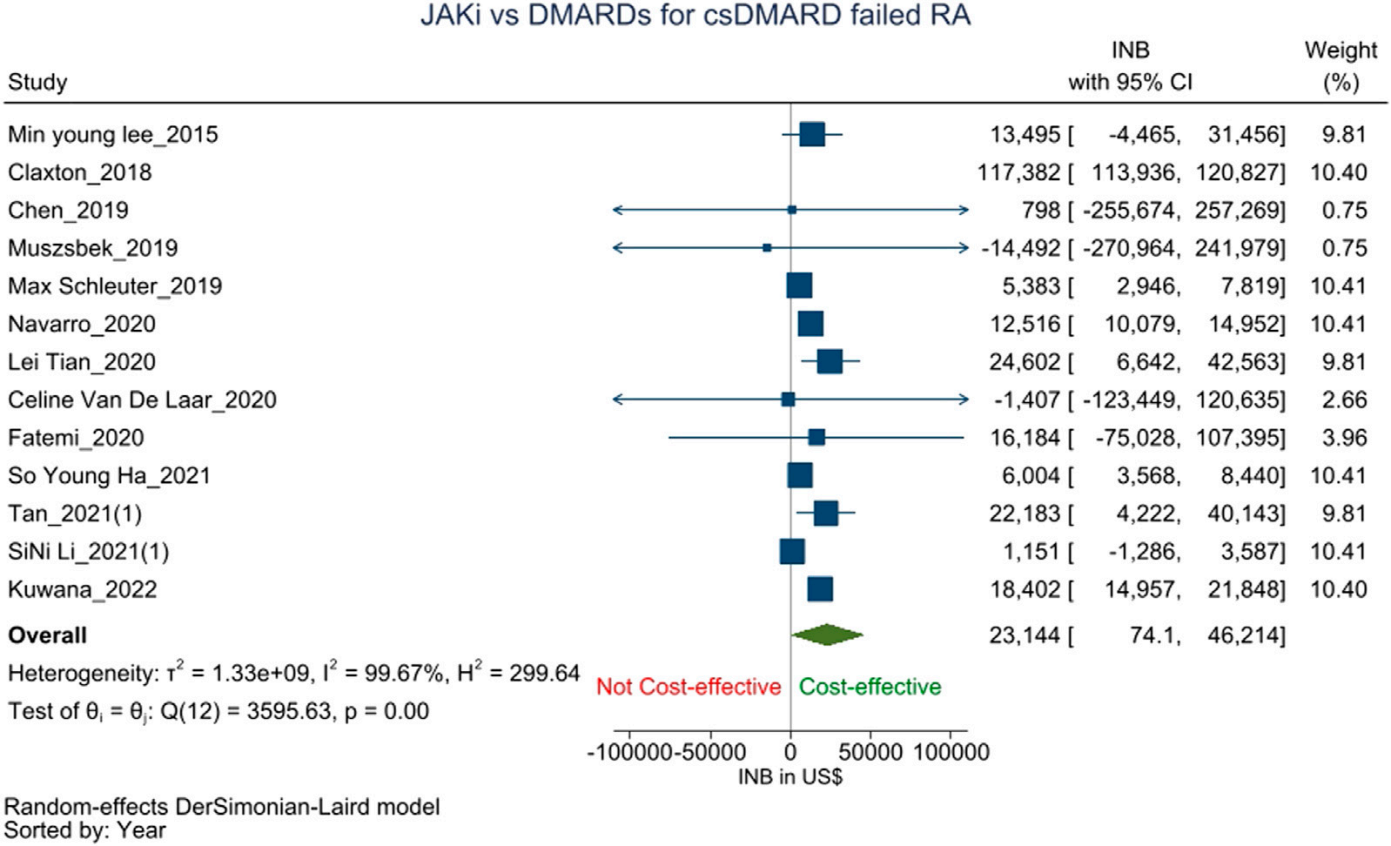
Forest plot of meta analysis showing pooled INBs with 95 percent CI values for JAK-i compared with csDMARDs/bDMARDs for csDMARD failed RA patients.

Thirteen studies (Lee et al., 2015; Claxton et al., 2018; Chen et al., 2019; Muszbek et al., 2019; Schlueter et al., 2019; Navarro et al., 2020; Tian et al., 2020; Van De Laar et al., 2020; Li et al., 2021a; Fatemi et al., 2021; Ha et al., 2021; Tan et al., 2021; Kuwana et al., 2022), assessed the cost-effectiveness of JAK-i *versus* csDMARDs (*n* = 2), TNF-a-i (*n* = 10) or IL-6-i (*n* = 1) as the second-line treatment for csDMARD failed RA patients. The pooled INB from these studies was $23,144 (74.1–46,214) with high heterogeneity (I^2^ = 99.67%), showing that JAK-i is cost-effective than csDMARDs/bDMARDs as the second-line treatment for csDMARD failed RA patients (Figure 2).

### Cost-effectiveness of JAK-i *versus* TNF-a-i as second line treatment for csDMARD failed RA

In a seperate analysis, studies which compared JAK-i *versus* TNF-a-i as second line treatment for csDMARD failed RA were pooled. The results showed that JAK-i is not cost-effective than TNF-a-i (INBp = $25,813, -5,714 to 57,340) with high heterogeneity and I^2^ = 99.74% (Supplementary Figure S11). However, the leave-one-out analysis found that one outlier [Claxton et al., 2018 (25)] is influencing the overall result (Supplementary Figure S12), and omitting the study from the analysis makes the result cost-effective with an INBp $9,402 (3,690 to 15,115) (Supplementary Figure S13).

### Cost-effectiveness of JAK-i versus csDMARDs/bDMARDs as third-line treatment for TNF-a-i failed RA

**FIGURE 3:**
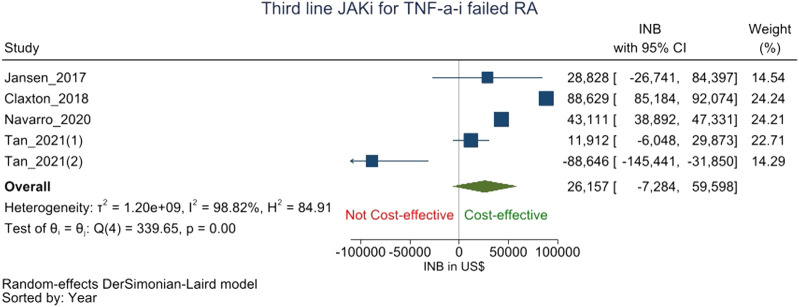
Forest plot of meta analysis showing pooled INBs with 95 percent CI values JAK-i compared with csDMARDs/bDMARDs for csDMARD-TNF-a-i failed RA patients.

JAK-i was compared to csDMARD/bDMARDs as the third-line treatment for RA patients who showed an inadequate response to TNF-a-i following csDMARD failure in five studies (Jansen et al., 2017; Claxton et al., 2018; Navarro et al., 2020; Tan et al., 2021; Tan et al., 2022). The pooled INB was $26,157 (-7,284 to 59,598) with high heterogeneity (I^2^ = 99.11%) which shows that JAK-i is not cost-effective to csDMARDs/bDMARD as a third-line treatment after cs-DMARD-TNF-a-i failure (Figure 3).

### Certainty of evidence- GRADE

The GRADE assessment revealed very low confidence in the overall findings and low confidence in separate analysis. The certainty of evidence from a lifetime horizon, societal perspective and HICs is low (Appendix 3).

## Discussion

We performed a systematic review and meta-analysis of published manuscripts in peer reviewed journals to synthesize the cost-effectiveness evidence of JAK-i for the treatment of moderate to severe RA. On overall comparison, JAK-i is cost-effective than other csDMARDs/bDMARDs but with high hetergeniety. As a second-line treatment, JAK-i is cost-effective than other csDMARDSs/bDMARDs for csDMARD-failed RA patients, but not cost-effective as a third line treatment for csDMARD-TNF-a-i failed RA patients.

Our observations showed a high degree of heterogeneity, which the sub-group analysis could not explain fully. The subgroup analysis based on the income classification of the countries found that the result is cost effective only for HICs and not in LMICs or UMICs. JAK-i is similarly cost-effective from a societal perspective but there are only four studies to support this.

In RA patients who had failed csDMARDs, JAK-i was more cost-effective than other csDMARDS/bDMARDs based on our meta-analysis. However, the results lose their robustness and JAK-i become not significantly cost-effective when we limit the comparator to TNF-a-i alone in a seperate analysis. Further, the leave-one-out analysis identified Claxton et al., 2019 (Claxton et al., 2018) as an outlier and pooling by omitting this study (Claxton et al., 2018), JAK-i turn out to be cost-effective than TNF-a-i, indicating the impact of an outlier.

In contrary to the findings of our meta-analysis, the individual studies which constituted our meta-analysis found that JAK-i is significantly cost-effective than TNF-a-i in RA patients who failed csDMARD. The reason being most of these studies reported cost-effectiveness based on ICER (point estimate) without considering any measures of dispersion whereas our meta-analysis reported pooled INB with measures of dispersion (95% CI) which may explain the discrepancy. The GRADE assessment also rated the certainty of the evidence to be low. Therefore, future studies should consider including measures of dispersion in addition to ICER to increase the robustness of their findings.

Further, the monetary value of currencies was adjusted for inflation and purchasing power parity using the CPI and PPP index to get the pooled estimate for the most recent year. As a result, a few studies that had previously indicated JAK-i to be cost-effective were no longer found to be so, after adjusting for the inflation and PPP index.

Drug costs have been the main determinants of cost-effectiveness in most of these studies, while hospitalization costs and the likelihood of serious infections are the other two factors (Li et al., 2021a). Lower drug cost and oral route of administration make JAK-i more preferable than TNF-a-i. Given the higher costs of biologics, Claxton et al., 2018 (Claxton et al., 2018) have hypothesized that using JAK-i as a second or third-line treatment may be less expensive than using it as a fourth-line treatment following two TNF-i failure. Similarly, corticosteroids, which are usually taken in conjunction with DMARDs, are less expensive and beneficial in reducing joint erosion and disease activity in RA. (Bae et al., 2003; Paglia et al., 2021) However, a recent study conducted in a real-world setting found that using an oral steroid concurrently did not improve the effectiveness of JAK inhibitors. (Iwamoto et al., 1478) EULAR also recommends using the lowest possible dose of oral steroids concomitant with bDMARDs/tDMARDs for the shortest time possible; (Smolen et al., 2022) hence, corticosteroids may only have a short-term effect on the cost and effectiveness of JAK-i.

According to clinical effectiveness data, JAK-i is not inferior to TNF-a-i in RA patients who have failed csDMARDs. (van Vollenhoven et al., 2012; Strand et al., 2016; Fleischmann et al., 2017; Taylor et al., 2017; Ren et al., 2018; Uttley et al., 2018) Based on National institute for health and care excellence (NICE)’s report, both TOFA and BARI are equally effective as other bDMARDs at treating moderate to severe RA, when used alone or in combination with MTX. (Baricitinib for moderate to severe, 2017; Tofacitinib for moderate to severe, 2017; Ren et al., 2018; Uttley et al., 2018) However, they are considered to be cost-effective options only for csDMARD IR severe RA patients and not for moderate RA. In bDMARD-IR severe RA patients, TOFA + MTX is cost-effective only when rituximab is contraindicated or not tolerated. (Baricitinib for moderate to severe, 2017; Tofacitinib for moderate to severe, 2017; Ren et al., 2018; Uttley et al., 2018) Further, JAK-i is more frequently linked to serious adverse events, including malignancy and cardiovascular disease (Venetsanopoulou et al., 2022). According to a recent study by Ytterberg et al. (2022), JAK-i is associated with a higher risk of serious infections, blood clots, cancer, and cardiovascular conditions than TNF inhibitors. Based on the study, the european medicines agency (EMA) advised restricting the use of JAK-i in patients above 65 years of age, those at increased risk of serious cardiovascular issues, those who smoke or have smoked for a significant period of time in the past, and those who are at increased risk of cancer. (Meeting highlights from the Pharmacovigilance, 2022) The Food and drug administration (FDA) previously came into a similar conclusion regarding an elevated risk of blood clots and death caused by JAK-i. (Xeljanz, 2021) As a result, the FDA mandated the boxed warning about the risks of fatal blood clots, cancer, severe heart-related events, and death. (FDA, 2021).

Several limitations should be noted when interpreting our conclusions. Most of the included studies were from HICs, while very few were from LMICs or UMICs and none from lower-income countries (LICs). Therefore, the results cannot be generalized to LICs, which warrants the need for cost-utility studies in the LICs setting. The majority of the included studies are model-based that assess the cost-effectiveness of treatment sequences in which JAK-i is one of the treatments in the second, third, or fourth position. As a result, rather than the costs and effectiveness of an individual drug, these studies reported the costs and effectiveness of the treatment sequence. Similarly, no CUA studies on other JAK-i such as UPA and FILG were found in systematic search. Most of the studies were undertaken from the perspective of the payer or health system with different discounting rates for costs and consequences. RA being a chronic condition, patients suffer high indirect medical and non-medical expenses. Hence, more research that considers these costs from a societal perspective is required.

## Conclusion

Meta-analysis suggests that JAK-I is cost-effective when used after csDMARD failure but not cost-effective when used after csDMARD-TNF-a-i failure with low certainty of evidence.

## Data Availability

The original contributions presented in the study are included in the article/Supplementary Material, further inquiries can be directed to the corresponding author.

## References

[B1] Adarkwah C. C. v. G. P. Hiligsmann M. Evers S. M. A. A Risk of bias in model-based economic evaluations: The ECOBIAS checklist. Expert Rev. pharmacoecon. Outcomes Res 2016;16(4):513–523. 10.1586/14737167.2015.1103185 26588001

[B2] Bae S. C. Corzillius M. Kuntz K. M. Liang M. H. (2003). Cost effectiveness of low dose corticosteroids versus non steroidal anti inflammatory drugs and COX 2 specific inhibitors in the long term treatment of rheumatoid arthritis. Rheumatology 42 (1), 46–53. 10.1093/rheumatology/keg029 12509612

[B3] Bagepally B. S. Chaikledkaew U. Chaiyakunapruk N. Attia J. Thakkinstian A. (2022). Meta-analysis of economic evaluation studies: Data harmonisation and methodological issues. BMC Health Serv. Res. 22 (1), 202. 10.1186/s12913-022-07595-1 35168619 PMC8845252

[B4] Bagepally B. S. Chaikledkaew U. Gurav Y. K. Anothaisintawee T. Youngkong S. Chaiyakunapruk N. (2020). Glucagon-like peptide 1 agonists for treatment of patients with type 2 diabetes who fail metformin monotherapy: Systematic review and meta-analysis of economic evaluation studies. BMJ Open Diabetes Res. Care 8 (1), e001020 10.1136/bmjdrc-2019-001020 PMC7371226 32690574

[B5] Bagepally B. S. Gurav Y. K. Anothaisintawee T. Youngkong S. Chaikledkaew U. Thakkinstian A. (2019). Cost utility of sodium-glucose cotransporter 2 inhibitors in the treatment of metformin monotherapy failed type 2 diabetes patients: A systematic review and meta-analysis. Value Health 22 (12), 1458–1469. 10.1016/j.jval.2019.09.2750 31806203

[B6] Baricitinib for moderate to severe rheumatoid arthritis. National Institute for Health and Care Excellence; 2017. Available from: https://www.nice.org.uk/guidance/ta466. London UK.

[B7] Chen D. Y. Hsu P. N. Tang C. H. Claxton L. Valluri S. Gerber R. A. (2019). Tofacitinib in the treatment of moderate-to-severe rheumatoid arthritis: A cost-effectiveness analysis compared with adalimumab in taiwan. J. Med. Econ. 22 (8), 777–787. 10.1080/13696998.2019.1606813 30982378

[B8] Claxton L. Taylor M. Gerber R. A. Gruben D. Moynagh D. Singh A. (2018). Modelling the cost-effectiveness of tofacitinib for the treatment of rheumatoid arthritis in the United States. Curr. Med. Res. Opin. 34 (11), 1991–2000. 10.1080/03007995.2018.1497957 29976110

[B9] Corporation. M (2018). Microsoft Excel [internet]. Available from: https://office.microsoft.com/excel.

[B10] Fatemi B. Rezaei S. Taheri S. Peiravian F. (2021). Cost-effectiveness analysis of tofacitinib compared with adalimumab and etanercept in the treatment of severe active rheumatoid arthritis; Iranian experience. Expert Rev. pharmacoecon. Outcomes Res. 21 (4), 775–784. 10.1080/14737167.2021.1834384 33043757

[B11] Fda (2021). FDA requires warnings about increased risk of serious heart-related events, cancer, blood clots, and death for JAK inhibitors that treat certain chronic inflammatory conditions. Food drug Adm. Drug Safety and Availability, 89 Available from: https://www.fda.gov/drugs/drug-safety-and-availability/fda-requires-warnings-about-increased-risk-serious-heart-related-events-cancer-blood-clots-and-death.

[B12] Fleischmann R. Mysler E. Hall S. Kivitz A. J. Moots R. J. Luo Z. (2017). Efficacy and safety of tofacitinib monotherapy, tofacitinib with methotrexate, and adalimumab with methotrexate in patients with rheumatoid arthritis (ORAL strategy): A phase 3b/4, double-blind, head-to-head, randomised controlled trial. Lancet 390 (10093), 457–468. 10.1016/s0140-6736(17)31618-5 28629665

[B13] Fournier M. Chen C. I. Kuznik A. Proudfoot C. Mallya U. G. Michaud K. (2019). Sarilumab monotherapy compared with adalimumab monotherapy for the treatment of moderately to severely active rheumatoid arthritis: An analysis of incremental cost per effectively treated patient. Clin. Outcomes Res. 11, 117–128. 10.2147/CEOR.S183076 PMC6368117 30787625

[B14] Grimm S. E. Wijnen B. Riemsma R. Fayter D. Armstrong N. Ahmadu C. (2021). Filgotinib for moderate to severe rheumatoid arthritis: An evidence review group perspective of a NICE single Technology appraisal. PharmacoEconomics 39 (12), 1397–1410. 10.1007/s40273-021-01080-z 34448148 PMC8599377

[B15] Guyatt G. Oxman A. D. Akl E. A. Kunz R. Vist G. Brozek J. (2011). GRADE guidelines: 1. Introduction—GRADE evidence profiles and summary of findings tables. J. Clin. Epidemiol. 64 (4), 383–394. 10.1016/j.jclinepi.2010.04.026 21195583

[B16] Ha S. Y. Shim Y. B. Lee M. Y. Koo B. S. Kim J. H. Jeon J. Y. (2021). Comparative cost-effectiveness of tofacitinib with continuing conventional synthetic disease-modifying anti-rheumatic drugs for active rheumatoid arthritis in South Korea. Rheumatol. Ther. 8 (1), 395–409. 10.1007/s40744-021-00278-z 33496958 PMC7991041

[B17] Hultcrantz M. Rind D. Akl E. A. Treweek S. Mustafa R. A. Iorio A. (2017). The GRADE Working Group clarifies the construct of certainty of evidence. J. Clin. Epidemiol. 87, 4–13. 10.1016/j.jclinepi.2017.05.006 28529184 PMC6542664

[B18] Iwamoto N. A-O. Sato S. Kurushima S. Michitsuji T. Nishihata S. Okamoto M. (1478). Real-world comparative effectiveness and safety of tofacitinib and baricitinib in patients with rheumatoid arthritis. Electron. Eng. 6362. 10.1186/s13075-021-02582-z PMC8299678 34301311

[B19] Jansen J. P. Incerti D. Mutebi A. Peneva D. MacEwan J. P. Stolshek B. (2017). Cost-effectiveness of sequenced treatment of rheumatoid arthritis with targeted immune modulators. J. Med. Econ. 20 (7), 703–714. 10.1080/13696998.2017.1307205 28294642

[B20] Kuwana M. Tamura N. Yasuda S. Fujio K. Shoji A. Yamaguchi H. (2022). Cost-effectiveness analyses of biologic and targeted synthetic disease-modifying anti-rheumatic diseases in patients with rheumatoid arthritis: Three approaches with a cohort simulation and real-world data. Mod. Rheumatol., 20, roac038. 10.1093/mr/roac038 35445720

[B21] Lau C. S. Chia F. Dans L. Harrison A. Hsieh T. Y. Jain R. (2018). 2018 update of the APLAR recommendations for treatment of rheumatoid arthritis Int. J. Rheum. Dis. 22 (3), 357–375. 10.1111/1756-185X.13513 30809944

[B22] Lee M. Y. Park S. K. Park S. Y. Byun J. H. Lee S. M. Ko S. K. (2015). Cost-effectiveness of tofacitinib in the treatment of moderate to severe rheumatoid arthritis in South Korea. Clin. Ther. 37 (8), 1662–1676. 10.1016/j.clinthera.2015.07.001 26243076

[B23] Li S. Li J. Peng L. Li Y. Wan X. (2021). Cost-effectiveness of baricitinib for patients with moderate-to-severe rheumatoid arthritis after methotrexate failed in China. Rheumatol. Ther. 8 (2), 863–876. 10.1007/s40744-021-00308-w 33893943 PMC8217482

[B24] Li S. Li J. Peng L. Li Y. Wan X. (2021). Cost-effectiveness of triple therapy vs. Biologic treatment sequence as first-line therapy for rheumatoid arthritis patients after methotrexate failure. Rheumatol. Ther. 8 (2), 775–791. 10.1007/s40744-021-00300-4 33772743 PMC8217385

[B25] Meeting highlights from the pharmacovigilance risk assessment committee (PRAC). South Amsterdam: European Medicines Agency; 2022, Available from: https://www.ema.europa.eu/en/news/meeting-highlights-pharmacovigilance-risk-assessment-committee-prac-24-27-october-2022.

[B26] Moher D. S. L. Prisma-P Group Clarke M. Ghersi D. Liberati A. Petticrew M. ,.Preferred reporting items for systematic review and meta-analysis protocols (PRISMA-P) 2015 statement. Syst. Rev. 2015;4(1):1. 10.1186/2046-4053-4-1 25554246 PMC4320440

[B27] Muszbek N. Proudfoot C. Fournier M. Chen C. I. Kuznik A. Kiss Z. (2019). Economic evaluation of sarilumab in the treatment of adult patients with moderately-to-severely active rheumatoid arthritis who have an inadequate response to conventional synthetic disease-modifying antirheumatic drugs. Adv. Ther. 36 (6), 1337–1357. 10.1007/s12325-019-00946-1 31004324 PMC6824456

[B28] Navarro F. Martinez-Sesmero J. M. Balsa A. Peral C. Montoro M. Valderrama M. (2020). Cost-effectiveness analysis of treatment sequences containing tofacitinib for the treatment of rheumatoid arthritis in Spain. Clin. Rheumatol. 39 (10), 2919–2930. 10.1007/s10067-020-05087-3 32303858 PMC7497326

[B29] O’Mahony J. F. Naber S. K. Normand C. Sharp L. O'Leary J. J. de Kok I. M. C. M. (2015). Beware of kinked Frontiers: A systematic review of the choice of comparator strategies in cost-effectiveness analyses of human papillomavirus testing in cervical screening Value Health. 18 (7), 1138–1151. 10.1016/j.jval.2015.09.2939 26686801

[B30] Ouzzani M. H. H. Fedorowicz Z. Elmagarmid A Rayyan-a web and mobile app for systematic reviews. Syst. Rev 2016;5(1):210. 10.1186/s13643-016-0384-4 27919275 PMC5139140

[B31] Paglia M. D. G. Silva M. T. Lopes L. C. Barberato-Filho S. Mazzei L. G. Abe F. C. (2021). Use of corticoids and non-steroidal anti-inflammatories in the treatment of rheumatoid arthritis: Systematic review and network meta-analysis. PLOS ONE 16 (4), e0248866. 10.1371/journal.pone.0248866 33826610 PMC8026036

[B32] Paulden M. (2020). Why it's time to abandon the ICER. Pharmacoeconomics 38 (8), 781–784. 10.1007/s40273-020-00915-5 32390066

[B33] Registry C. 2021. Center for the evaluation of value and risk in health. Available from: https://cevr.tuftsmedicalcenter.org/databases/cea-registry.

[B34] Ren S. Bermejo I. Simpson E. Wong R. Scott D. L. Young A. (2018). Baricitinib for previously treated moderate or severe rheumatoid arthritis: An evidence review group perspective of a NICE single Technology appraisal. PharmacoEconomics 36 (7), 769–778. 10.1007/s40273-018-0616-7 29502174 PMC5999127

[B35] Rohatgi A. (2021). WebPlotDigitizer. USA. Available from: https://automeris.io/WebPlotDigitizer.

[B36] Russell L. B. Gold M. R. Siegel J. E. Daniels N. Weinstein M. C. (1996). The role of cost-effectiveness analysis in health and medicine. Panel on Cost-Effectiveness in Health and Medicine. JAMA J. Am. Med. Assoc. 276 (14), 1172–1177. 10.1001/jama.276.14.1172 8827972

[B37] Schlueter M. Finn E. Diaz S. Dilla T. Inciarte-Mundo J. Fakhouri W. (2019). Cost-effectiveness analysis of baricitinib versus adalimumab for the treatment of moderate-to-severe rheumatoid arthritis in Spain. Clin. Outcomes Res. 11, 395–403. 10.2147/CEOR.S201621 PMC6560251 31239736

[B38] Siegel J. E. Weinstein M. C. Russell L. B. Gold M. R. (1996). Recommendations for reporting cost-effectiveness analyses. Panel on cost-effectiveness in health and medicine. JAMA 276 (16), 1339–1341. 10.1001/jama.276.16.1339 8861994

[B39] Smolen J. A-O. Landewé R. A-O. Bergstra S. A-O. Kerschbaumer A. A-O. Sepriano A. A-O. Aletaha D. A-O. (2022). EULAR recommendations for the management of rheumatoid arthritis with synthetic and biological disease-modifying antirheumatic drugs: 2022 update. Electron. Eng., 56 1468–2060. 10.1136/ard-2022-223356

[B40] Smolen J. S. Aletaha D. McInnes I. B. (2016). Rheumatoid arthritis. Lancet 388 (10055), 2023–2038. 10.1016/S0140-6736(16)30173-8 27156434

[B41] Smolen J. S. Landewe R. B. M. Bijlsma J. W. J. Burmester G. R. Dougados M. Kerschbaumer A. (2020). EULAR recommendations for the management of rheumatoid arthritis with synthetic and biological disease-modifying antirheumatic drugs: 2019 update. Ann. Rheum. Dis. 79 (6), 685–699. 10.1136/annrheumdis-2019-216655 31969328

[B42] StataCorp (2019).Stata statistical software: Release 17. 17. College Station, TX: StataCorp LLC Available from: https://www.stata.com/.

[B43] Strand V. van Vollenhoven R. F. Lee E. B. Fleischmann R. Zwillich S. H. Gruben D. (2016). Tofacitinib or adalimumab versus placebo: Patient-reported outcomes from a phase 3 study of active rheumatoid arthritis. Rheumatol. Oxf. 55 (6), 1031–1041. 10.1093/rheumatology/kev442 PMC4870388 26929445

[B44] Tan C. Li S. Yi L. Zeng X. Peng L. Qin S. (2021). Tofacitinib in the treatment of moderate-to-severe rheumatoid arthritis in China: A cost-effectiveness analysis based on a mapping algorithm derived from a Chinese population. Adv. Ther. 38 (5), 2571–2585. 10.1007/s12325-021-01733-7 33837917

[B45] Tan C. Luo X. Li S. Yi L. Zeng X. Peng L. (2022). Sequences of biological treatments for patients with moderate-to-severe rheumatoid arthritis in the era of treat-to-target in China: A cost-effectiveness analysis. Clin. Rheumatol. 41 (1), 63–73. 10.1007/s10067-021-05876-4 34373933

[B46] Taylor P. C. Keystone E. C. van der Heijde D. Weinblatt M. E. Del Carmen Morales L. Reyes Gonzaga J. (2017). Baricitinib versus placebo or adalimumab in rheumatoid arthritis. N. Engl. J. Med. 376 (7), 652–662. 10.1056/NEJMoa1608345 28199814

[B47] Tian L. Xiong X. Guo Q. Chen Y. Wang L. Dong P. (2020). Cost-effectiveness of tofacitinib for patients with moderate-to-severe rheumatoid arthritis in China. Pharmacoeconomics 38 (12), 1345–1358. 10.1007/s40273-020-00961-z 32929677

[B48] Tofacitinib for moderate to severe rheumatoid arthritis. National Institute for Health and Care Excellence; 2017. Available from: https://www.nice.org.uk/guidance/ta480. London, UK.

[B49] Uttley L. Bermejo I. Ren S. Martyn-St James M. Wong R. Scott D. L. (2018). Tofacitinib for treating rheumatoid arthritis after the failure of disease-modifying anti-rheumatic drugs: An evidence review group perspective of a NICE single Technology appraisal. Pharmacoeconomics 36 (9), 1063–1072. 10.1007/s40273-018-0639-0 29546668

[B50] Van De Laar C. J. Oude Voshaar M. A. H. Fakhouri W. K. H. Zaremba-Pechmann L. De Leonardis F. De La Torre I. (2020). Cost-effectiveness of a JAK1/JAK2 inhibitor vs a biologic disease-modifying antirheumatic drug (bDMARD) in a treat-to-target strategy for rheumatoid arthritis. Clin. Outcomes Res. 12, 213–222. 10.2147/CEOR.S231558 PMC7167259 32346301

[B51] van Vollenhoven R. F. Fleischmann R. Cohen S. Lee E. B. García Meijide J. A. Wagner S. (2012). Tofacitinib or adalimumab versus placebo in rheumatoid arthritis. N. Engl. J. Med. 367 (6), 508–519. 10.1056/NEJMoa1112072 22873531

[B52] Venetsanopoulou A. I. Voulgari P. V. Drosos A. A. (2022). Janus kinase versus TNF inhibitors: Where we stand today in rheumatoid arthritis. Expert Rev. Clin. Immunol. 18 (5), 485–493. 10.1080/1744666X.2022.2064275 35535405

[B53] World Economic Outlook Database (2021). International monetary fund. New York, NY. Fund IM.

[B54] Xeljanz X. R. (2021). Initial safety trial results find increased risk of serious heart-related problems and cancer with arthritis and ulcerative colitis medicine Xeljanz (tofacitinib). Food drug Adm. 2021. https://www.fda.gov/drugs/drug-safety-and-availability/initial-safety-trial-results-find-increased-risk-serious-heart-related-problems-and-cancer-arthritis.

[B55] Ytterberg S. R. Bhatt D. L. Mikuls T. R. Koch G. G. Fleischmann R. Rivas J. L. (2022). Cardiovascular and cancer risk with tofacitinib in rheumatoid arthritis. N. Engl. J. Med. 386 (4), 316–326. 10.1056/NEJMoa2109927 35081280

