# Supplementary material for: Cost-effectiveness of janus kinase inhibitors for rheumatoid arthritis: A systematic review and meta-analysis of cost-utility studies
Source: Front Pharmacol. 2022 Dec 13;13:1090361. doi: 10.3389/fphar.2022.1090361 (PMC9792993; doi:10.3389/fphar.2022.1090361)
Supplement: Supplementary file 1 [file DataSheet1.PDF]

Supplementary Material

Of manuscript, Sajith Kumar S *Ph.D.*, Madhumitha Haridoss *Ph.D.*, Bhavani Shankara Bagepally *MBBS, Ph.D., M.Sc.* ***Cost-effectiveness of Janus Kinase inhibitors for Rheumatoid Arthritis: A systematic review and meta-analysis of cost-utility studies***

Supplementary Figures: Page 2 to 14

Supplementary Figure 1- Assessment of Risk of Bias using ECOBIAS Checklist.....2
Supplementary Figure 2- Pooled INBs for JAKi vs csDMARDs/bDMARDS.....3
Supplementary Figure 3- Leave one out analysis for JAKi vs csDMARDs/bDMARDS.....4
Supplementary Figure 4- Galbraith plot for JAKi vs csDMARDs/bDMARDS.....5
Supplementary Figure 5- Funnel plot for JAKi vs csDMARDs/bDMARDS.....6
Supplementary Figure 6- Subgroup analysis of pooled INBs based on study perspectives.....7
Supplementary Figure 7- Subgroup analysis of Pooled INBs based on Income classification.....8
Supplementary Figure 8- Subgroup analysis of pooled INBs based on threshold.....9
Supplementary Figure 9- Subgroup analysis of pooled INBs based on scenario.....10
Supplementary Figure 10- Subgroup analysis of pooled INBs based on time horizon.....11
Supplementary Figure 11- Second line JAKi vs TNF-a-i for csDMARD failed RA.....12
Supplementary Figure 12- Leave one out analysis for JAKi vs TNF-a-i for csDMARD failed RA.....13
Supplementary Figure 13- Leave one out analysis for JAKi vs TNFa-i in csDMARD failure patients after removing the outlier (Claxton, 2018).....14

Appendices: page 15 to 24

Appendix I: Search Strategy.....15
Appendix II: Methods.....19
Appendix III: Summary of Findings of GRADE Assessment.....22
Appendix IV: PRISMA Checklist for Abstract.....23
Appendix V: PRISMA checklist.....24

Supplementary Figure 1- Assessment of Risk of Bias using ECOBIAS Checklist

**Issue Addressed**	**Author_ Year**	**Fournier _2019**	**Chen_2019**	**Navarro _2020**	**Lei Tian _2020**	**Van De Laar_2020**	**Muszsbek _2019**	**Min young lee_2015**	**So Young Ha_2021**	**Schleuter _2019**	**Jansen _2017**	**Claxton _2018**	**Fatemi _2020**	**Tan _2021(1)**	**Tan _2021(2)**	**SiniLI _2021 (1)**	**SiniLI _2021 (2)**	**Kuwana _2022**
Narrow perspective bias		P	Y	Y	Y	Y	Y	Y	Y	Y	Y	Y	Y	Y	Y	Y	Y	Y
Inefficient comparator bias		Y	Y	Y	Y	Y	Y	Y	Y	Y	Y	Y	Y	Y	Y	Y	Y	Y
Cost measurement omission bias		P	Y	P	Y	P	Y	Y	Y	Y	Y	Y	Y	Y	Y	Y	Y	P
Intermittent data collection bias		Y	Y	P	Y	P	Y	Y	Y	P	Y	Y	Y	Y	Y	Y	Y	P
Invalid valuation bias		P	Y	P	Y	P	Y	Y	Y	P	Y	P	Y	Y	Y	P	Y	P
Ordinal ICER bias		Y	Y	P	P	U	Y	Y	Y	P	Y	P	Y	Y	U	P	Y	P
Double-counting bias		P	P	U	Y	U	Y	P	Y	P	Y	P	P	Y	Y	U	U	U
Inappropriate discounting bias		Y	Y	Y	Y	Y	Y	Y	Y	Y	Y	Y	Y	Y	Y	Y	Y	Y
Limited sensitivity analysis bias		U	P	P	P	U	Y	U	P	P	Y	P	Y	Y	P	Y	P	P
Sponsor bias		Y	Y	Y	Y	Y	Y	Y	Y	Y	Y	Y	Y	Y	Y	Y	Y	Y
Reporting and dissemination bias		U	U	U	U	U	Y	U	U	Y	Y	Y	U	Y	Y	Y	Y	Y
Structural assumptions bias		Y	Y	Y	Y	Y	Y	Y	Y	Y	Y	Y	Y	P	P	Y	Y	Y
No treatment comparator bias		Y	Y	Y	Y	Y	Y	Y	Y	Y	Y	Y	Y	Y	Y	Y	Y	Y
Wrong model bias		Y	Y	Y	Y	Y	Y	Y	Y	Y	Y	Y	Y	Y	Y	Y	Y	Y
Limited time horizon bias		P	Y	Y	Y	Y	Y	Y	Y	Y	Y	Y	Y	Y	Y	Y	Y	Y
Bias related to data identification		P	Y	P	Y	P	Y	U	P	P	Y	P	Y	Y	Y	Y	Y	Y
Bias related to baseline data		Y	Y	P	P	P	Y	U	P	P	P	P	P	P	P	P	Y	U
Bias related to treatment effects		P	Y	P	P	U	Y	P	P	P	Y	P	P	P	Y	Y	Y	P
Bias related to quality-of-life weights		P	Y	P	Y	P	Y	U	Y	Y	Y	Y	Y	Y	Y	P	Y	P
Non-transparent data incorporation bias		P	Y	P	Y	P	Y	Y	Y	Y	Y	Y	Y	P	Y	Y	Y	P
Limited scope bias		U	P	P	Y	P	Y	P	P	P	Y	Y	Y	U	Y	P	P	U
Bias related to internal consistency		U	U	U	U	U	U	U	U	U	U	U	U	U	U	U	U	U

Y- Yes, N-No, P-Partly, U-Unclear, NA- Not Applicable |

Source: http://dx.doi.org/10.1586/14737167.2015.1103185

Supplementary Figure 2- Pooled INBs for JAKi vs csDMARDs/bDMARDS

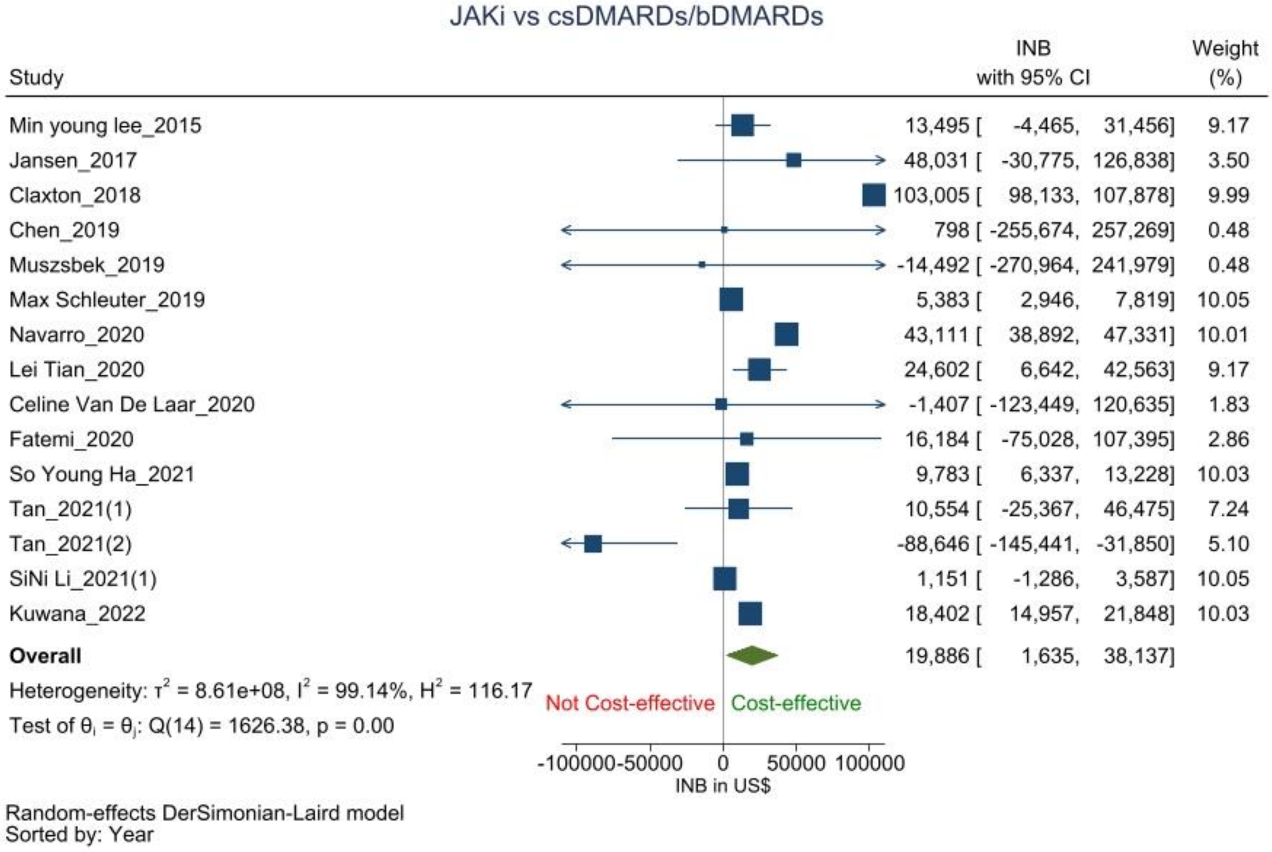

Supplementary Figure 3- Leave one out analysis for JAKi vs csDMARDs/bDMARDS

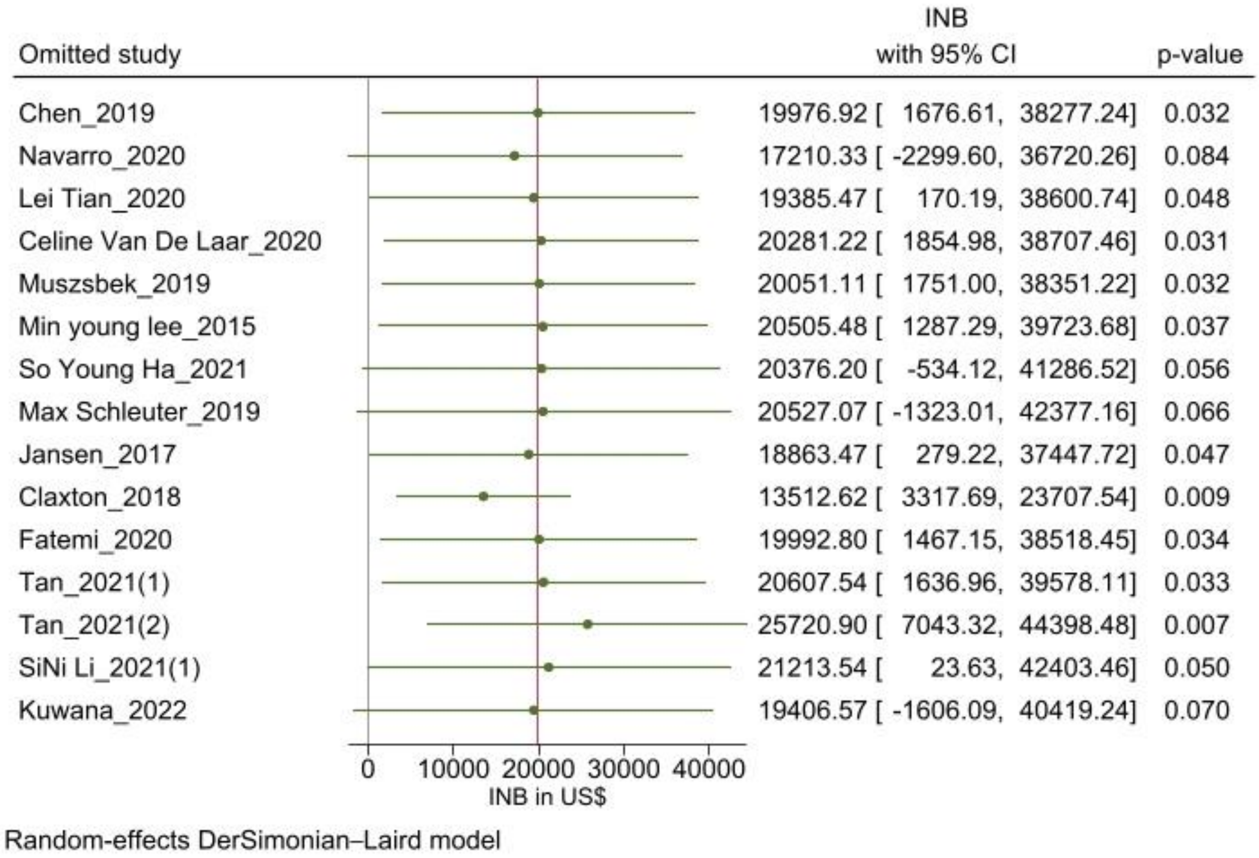

JAKi- Janus Kinase Inhibitor, csDMARD- conventional synthetic disease modifying anti rheumatic drugs, bDMARDs- biologic disease modifying anti rheumatic drugs, CI- confidence interval

Supplementary Figure 4- Galbraith plot for JAKi vs csDMARDs/bDMARDS

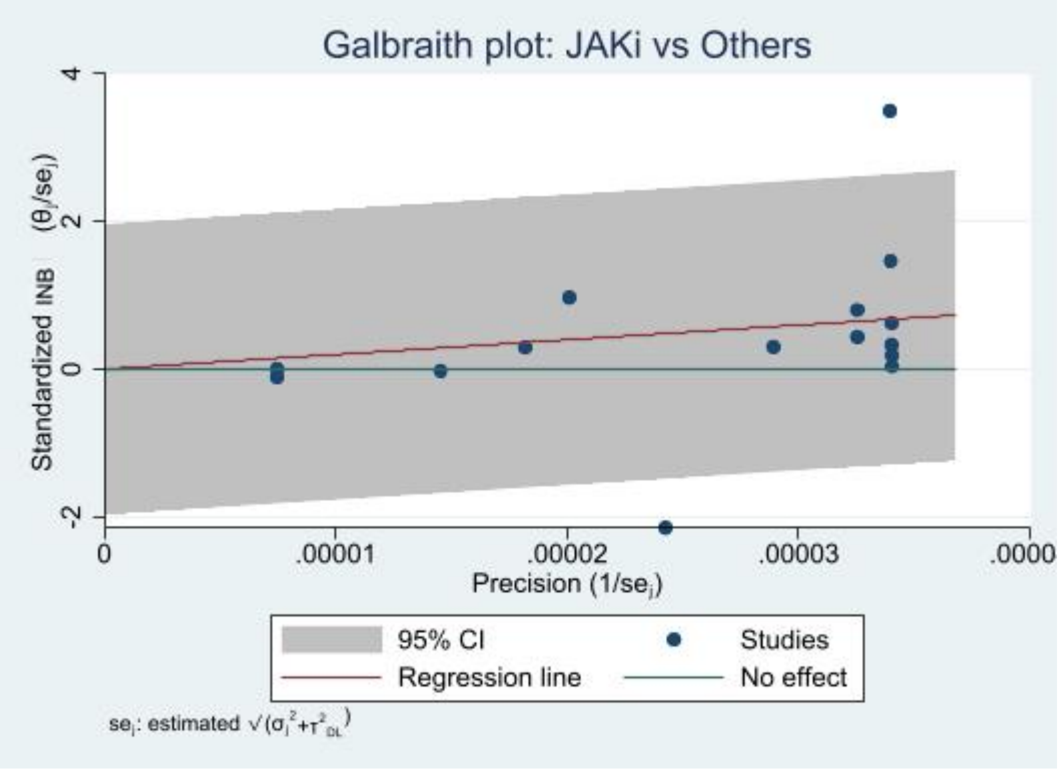

JAKi- Janus Kinase Inhibitor, csDMARD- conventional synthetic disease modifying anti rheumatic drugs, bDMARDs- biologic disease modifying anti rheumatic drugs, CI- confidence interval

Supplementary Figure 5- Funnel plot for JAKi vs csDMARDs/bDMARDS

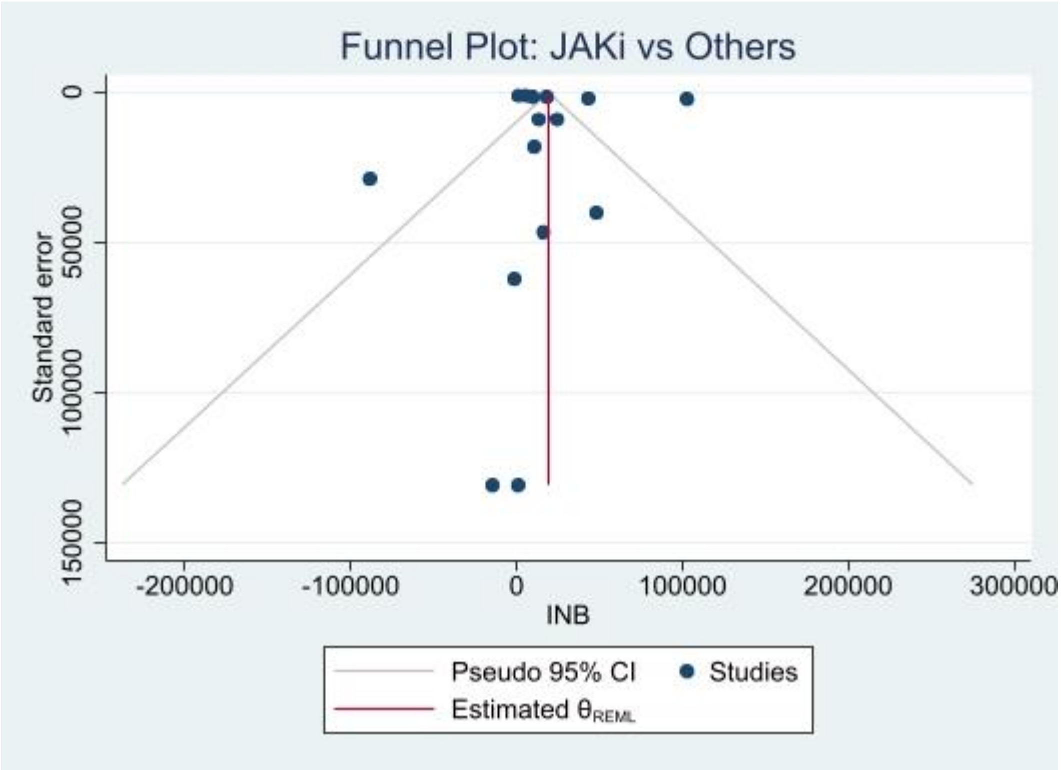

JAKi- Janus Kinase Inhibitor, csDMARD- conventional synthetic disease modifying anti rheumatic drugs, bDMARDs- biologic disease modifying anti rheumatic drugs, CI- confidence interval

Supplementary Figure 6- Subgroup analysis of pooled INBs based on study perspectives

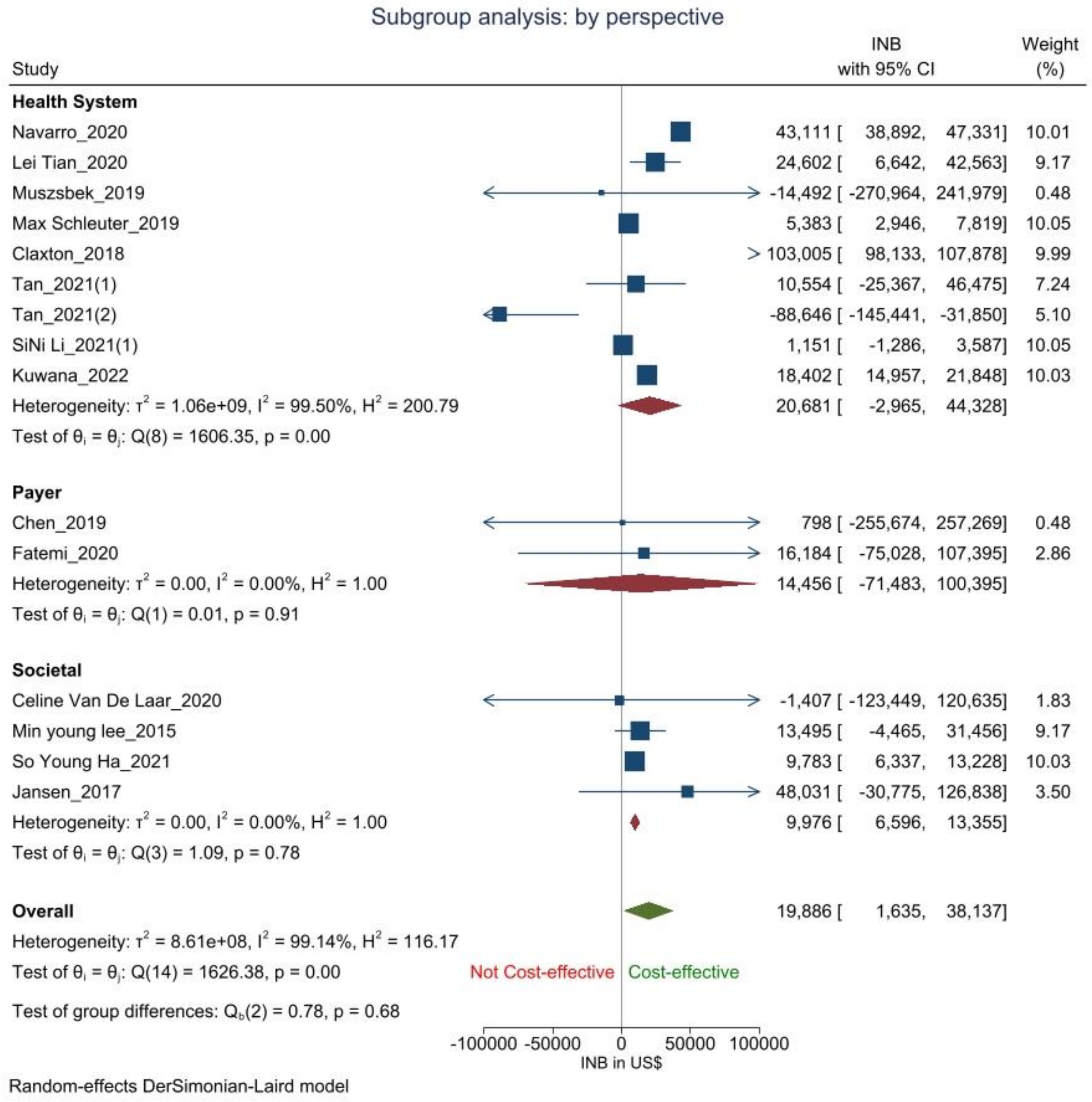

INB- incremental net benefit, CI- confidence interval

Supplementary Figure 7- Subgroup analysis of Pooled INBs based on Income classification

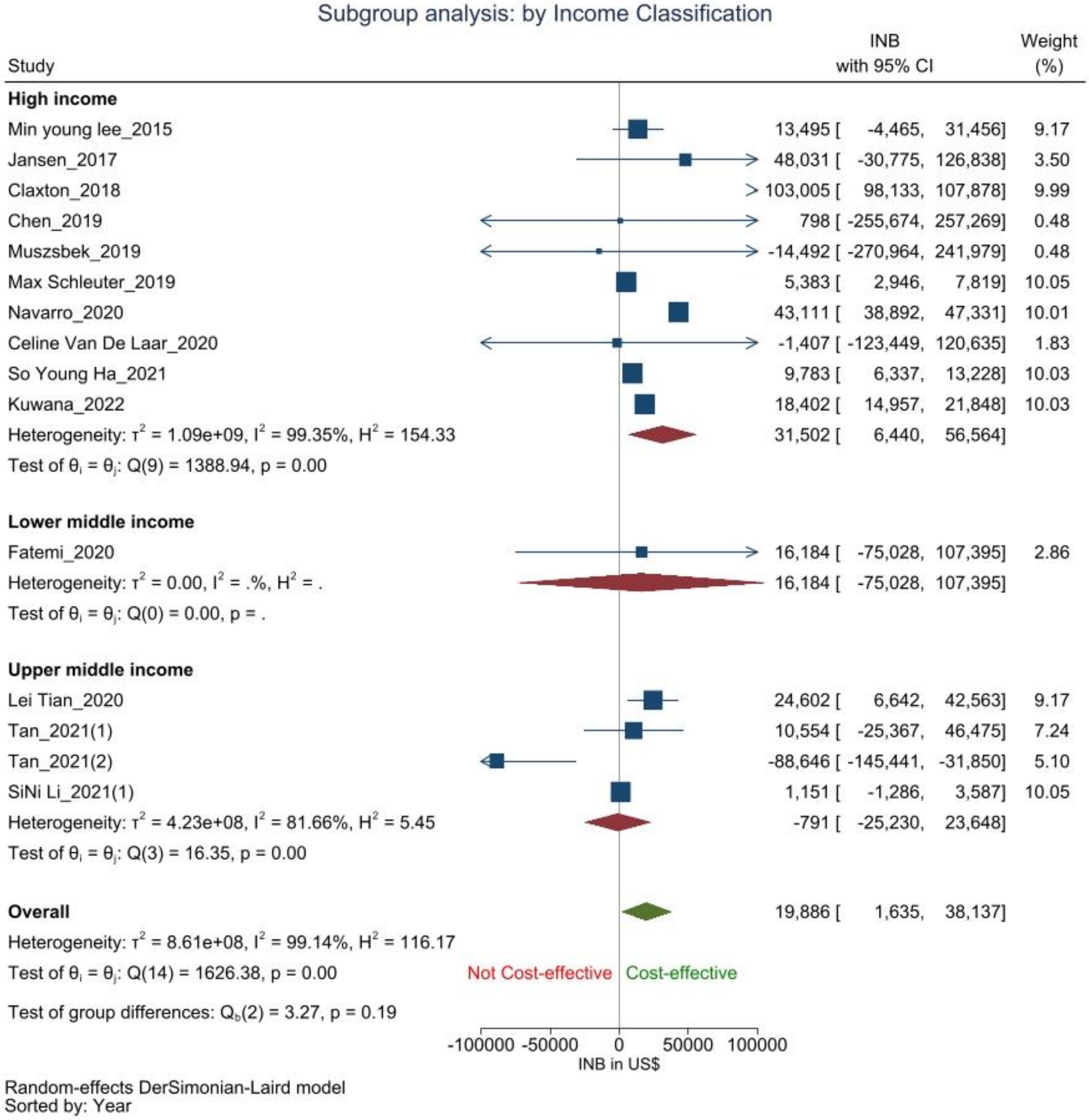

INB- incremental net benefit, CI- confidence interval

Supplementary Figure 8- Subgroup analysis of pooled INBs based on threshold

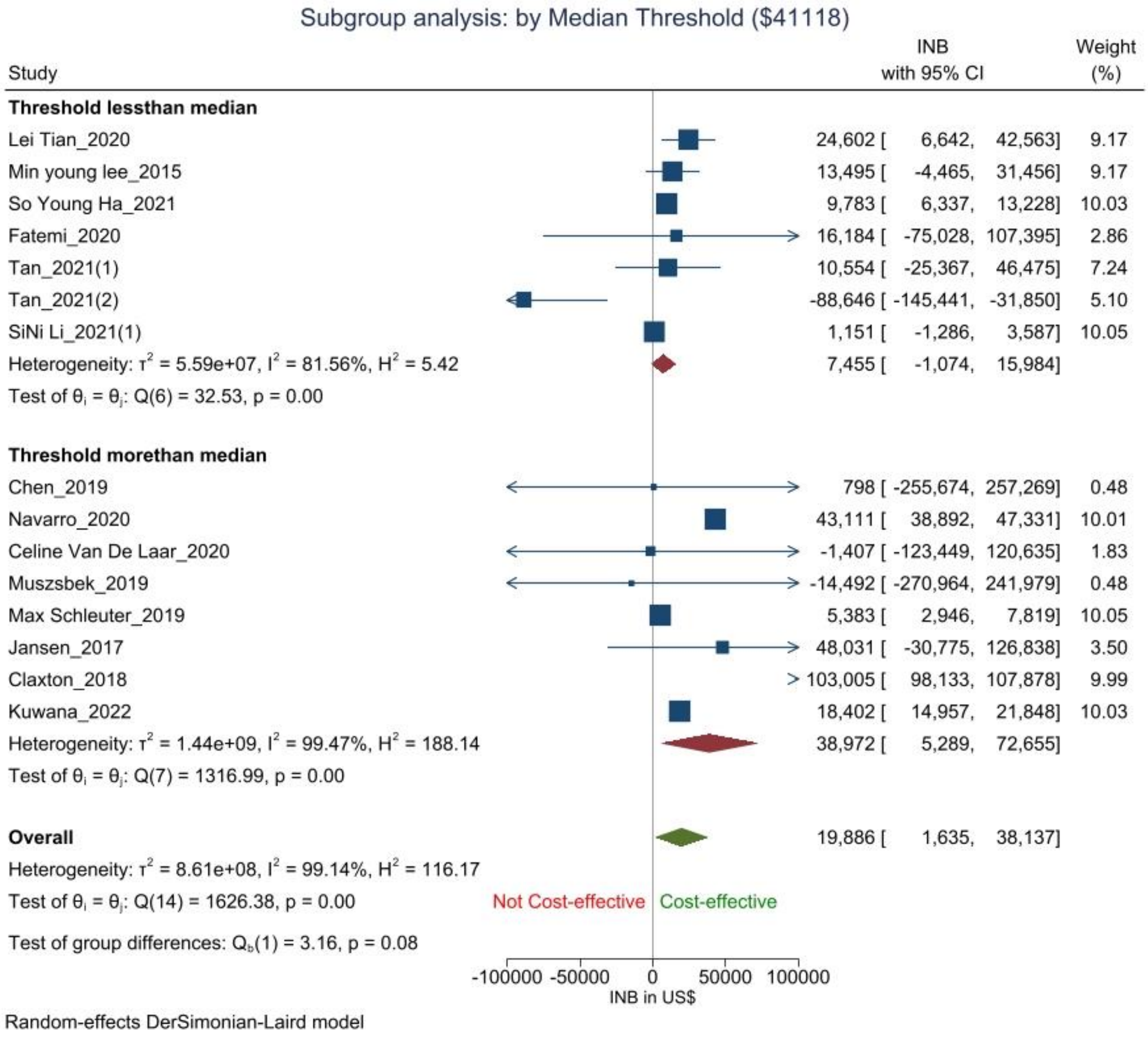

INB- incremental net benefit, CI- confidence interval

Supplementary Figure 9- Subgroup analysis of pooled INBs based on scenario

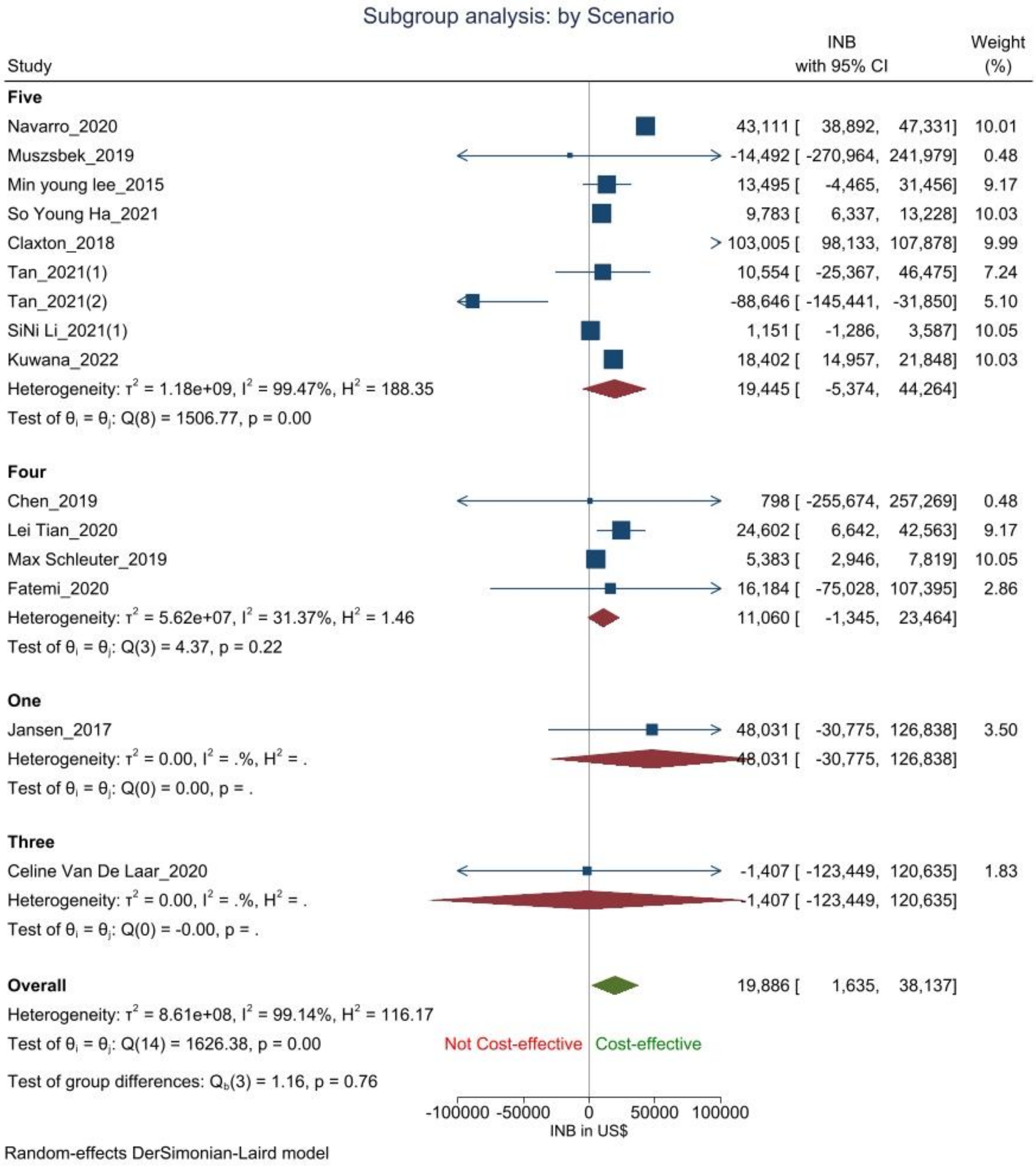

INB- incremental net benefit, CI- confidence interval

Supplementary Figure 10- Subgroup analysis of pooled INBs based on time horizon

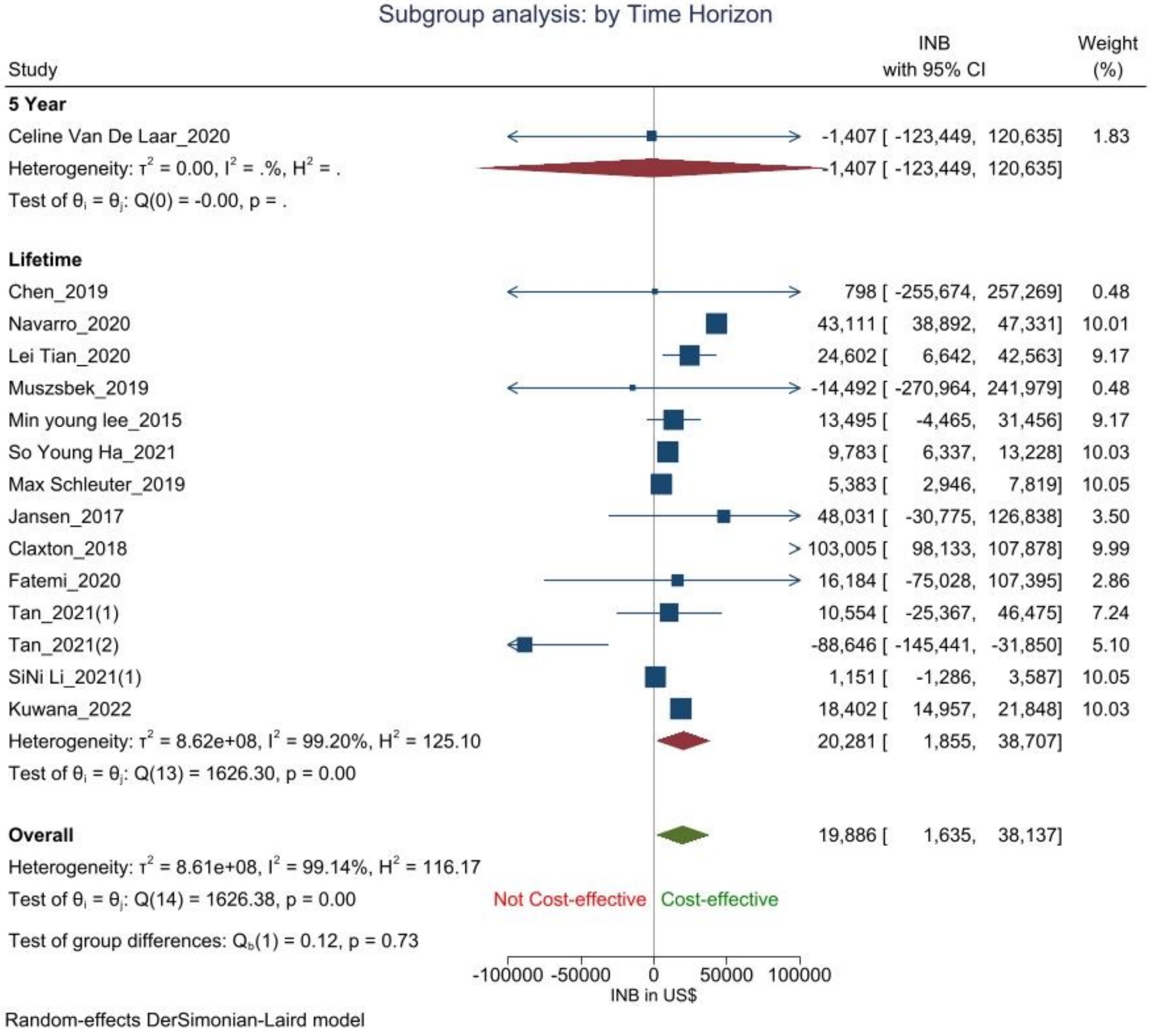

INB- incremental net benefit, CI- confidence interval

Supplementary Figure 11- Second line JAKi vs TNF-a-i for csDMARD failed RA

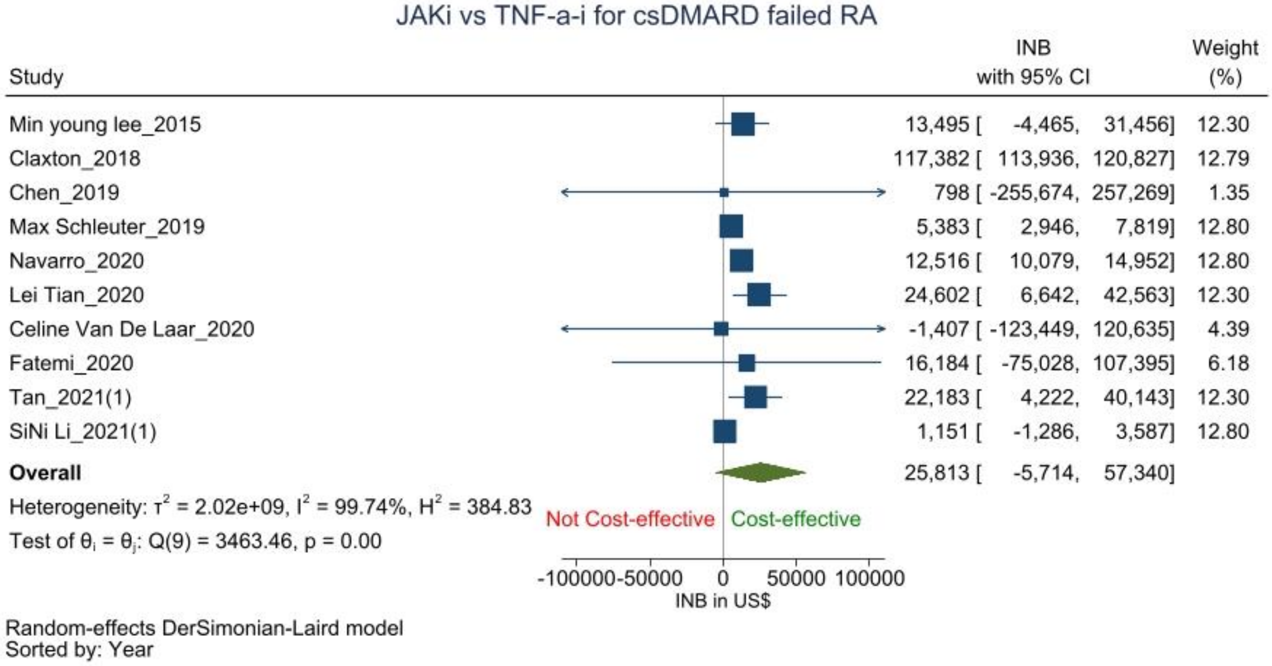

INB- incremental net benefit, JAKi- Janus Kinase Inhibitor, CI- confidence interval, DMARD- disease modifying anti rheumatic drugs, csDMARD- conventional synthetic disease modifying anti rheumatic drugs, RA- Rheumatoid Arthritis.

Supplementary Figure 12- Leave one out analysis for JAKi vs TNF-a-i for csDMARD failed RA

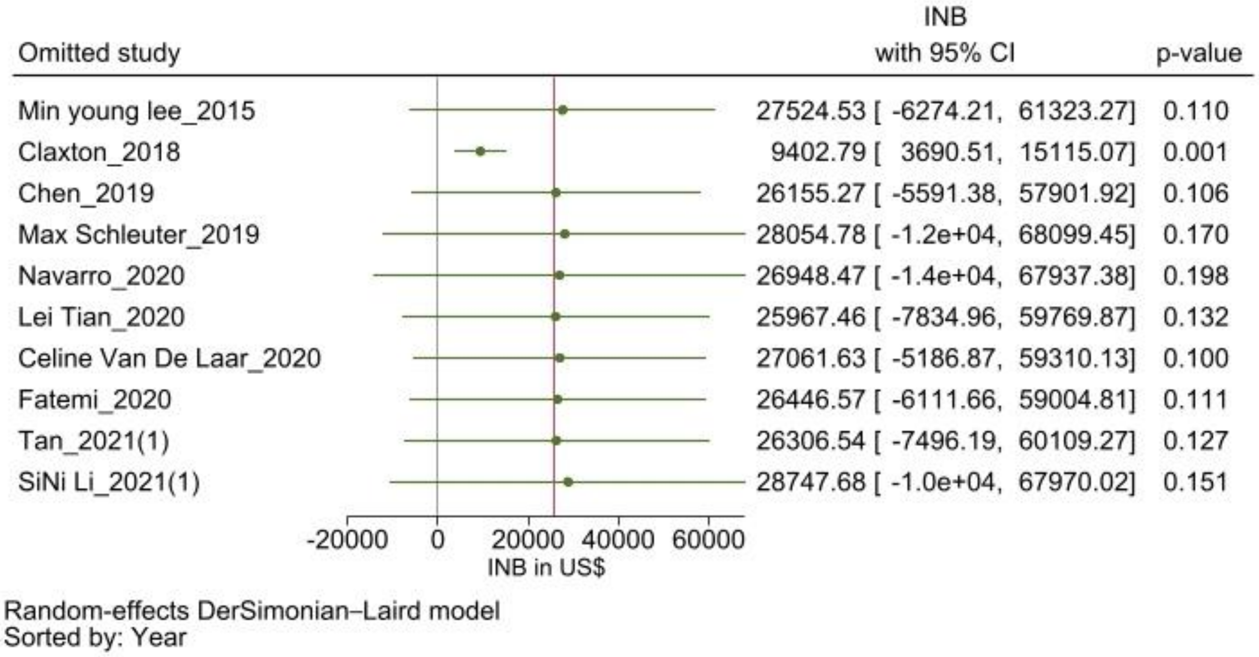

JAKi- Janus Kinase Inhibitor, CI- confidence interval, TNF-a-i - Tumor necrosis factor -alpha-inhibitors, csDMARD- conventional synthetic disease modifying anti rheumatic drugs, RA-Rheumatoid Arthritis.

Supplementary Figure 13- Leave one out analysis for JAKi vs TNFa-i in csDMARD failure patients after removing the outlier (Claxton, 2018)

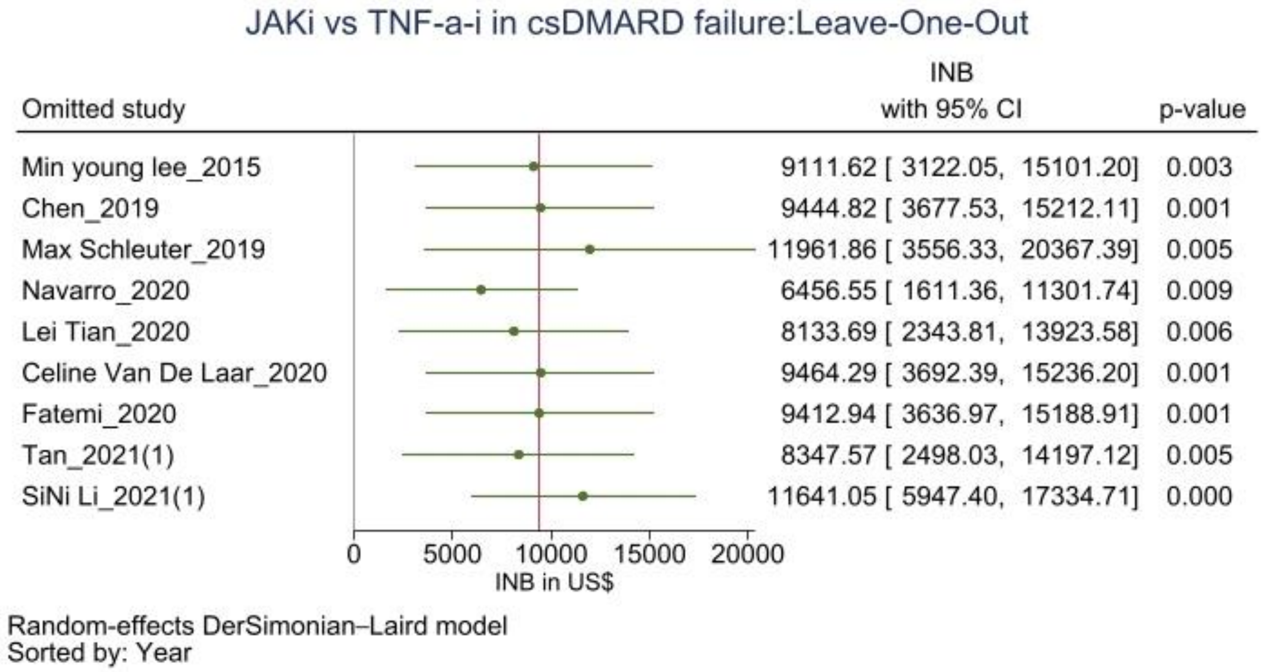

JAKi- Janus Kinase Inhibitor, CI- confidence interval, TNF-a-i - Tumor necrosis factor -alpha-inhibitors, csDMARD- conventional synthetic disease modifying anti rheumatic drugs.

Online Supplementary Material

Appendix I: Search Strategy

PICOS	PUBMED search terms	Hits on date 12th Feb 2021	Hits on date 5th May 2022
P	"arthritis, rheumatoid"[MeSH Terms] OR rheumatoid arthritis	153,377	161,308
I	tnf OR "Tumor Necrosis Factor" OR "JAK inhibitor" OR "JAK inhibitors" OR "Janus kinase inhibitor" OR DMARD OR "disease modifying anti-rheumatic drugs" OR biologics OR upadacitinib OR Rinvoq OR baricitinib OR Olumiant OR Simponi OR Simponi Aria OR golimumab OR certolizumab pegol OR certolizumab OR Inflectra OR infliximab-dyyb OR infliximab OR Remicade OR etanercept-szzs OR Erelzi OR etanercept OR Enbrel OR adalimumab-atto OR Amjevita OR adalimumab OR Humira OR Cyltezo OR Hyrimoz OR Cimzia OR methotrexate OR Amethopterin OR MTX OR Otrexup OR Trexall OR Rheumatrex OR Rasuvo OR tofacitinib OR Xeljanz OR Rituximab OR Rituxan OR Truxima OR Mabthera OR Ocrelizumab OR Ofatumumab OR Ublituximab	7,018,085	7,601,344
O	QALY OR “quality adjusted” OR “life year” OR “life years” OR DALY OR “disability adjusted” OR “cost effective” OR cost-utility OR “cost utility” OR ICER OR ICERS OR INB OR "economics"[MeSH Terms] OR "economics, pharmaceutical"[MeSH Terms]	703,048	743,921
PICS	PIO	1,353	1,455
	From 2021 to 5th May 2022		90

PICOS	Embase Search terms	Hits on date 12th Feb 2021	Hits on date 5th May 2022
P	'rheumatoid arthritis'/exp OR 'arthritis deformans' OR 'arthritis, rheumatoid' OR 'arthrosis deformans' OR 'beauvais disease' OR 'chronic articular rheumatism' OR 'chronic polyarthritis' OR 'chronic progressive poly arthritis' OR 'chronic progressive polyarthritis' OR 'chronic rheumatoid arthritis' OR 'disease, beauvais' OR 'inflammatory arthritis' OR 'polyarthritis, primary chronic' OR 'primary chronic polyarthritis' OR 'progressive polyarthritis, chronic' OR 'rheumarthritis' OR 'rheumatic arthritis' OR 'rheumatic polyarthritis' OR 'rheumatism, chronic articular' OR 'rheumatoid arthritis'	249,996	272,136
I	'tumor necrosis factor inhibitor'/exp OR 'tnf alpha inhibitor' OR 'tnf inhibitor' OR 'anti tnf agent' OR 'anti tnf alpha agent' OR 'anti tumor necrosis factor agent' OR 'anti tumour necrosis factor agent' OR 'tumor necrosis factor alpha inhibitor' OR 'tumor necrosis factor inhibitor' OR 'tumor necrosis factor inhibitors' OR 'tumour necrosis factor alpha inhibitor' OR 'tumour necrosis factor inhibitor' OR 'janus kinase inhibitor'/exp OR 'jak inhibitor' OR 'janus kinase inhibitor' OR 'janus kinase inhibitors' OR 'janus tyrosine kinase inhibitor' OR 'disease modifying antirheumatic drug'/exp OR 'disease modifying antirheumatic agent' OR 'disease modifying antirheumatic drug' OR 'disease modifying antirheumatic drugs' OR 'baricitinib'/exp OR 'baricitinib' OR 'olumiant' OR 'upadacitinib'/exp OR 'rinvoq' OR 'upadacitinib' OR 'upadacitinib 2, 3 dihydroxybutanedioate' OR 'upadacitinib hemihydrate' OR 'upadacitinib hydrate' OR 'upadacitinib tartrate' OR	356,629	396,592

	'golimumab'/exp OR 'golimumab' OR 'simponi' OR 'simponi aria' OR 'certolizumab pegol'/exp OR 'certolizumab pegol' OR 'cimzia' OR 'pegylated tumor necrosis factor alpha antibody fab fragment' OR 'pegylated tumour necrosis factor alpha antibody fab fragment' OR 'certolizumab'/exp OR 'etanercept'/exp OR 'avent' OR 'benepali' OR 'brenzys' OR 'embrel' OR 'enbrel' OR 'enerceptan' OR 'erelzi' OR 'etanercept' OR 'etanercept szzs' OR 'etanercept ykro' OR 'etanercept-szzs' OR 'etanercept-ykro' OR 'eticovo' OR 'infinitam' OR 'lifmior' OR 'nepexto' OR 'opinercept' OR 'recombinant tumor necrosis factor receptor fc fusion protein' OR 'recombinant tumour necrosis factor receptor fc fusion protein' OR 'tumor necrosis factor receptor fc fusion protein' OR 'tumour necrosis factor receptor fc fusion protein' OR 'tunex' OR 'tofacitinib'/exp OR 'tasocitinib' OR 'tasocitinib citrate' OR 'tofacitinib' OR 'tofacitinib citrate' OR 'xeljanz' OR 'xeljanz xr' OR 'adalimumab'/exp OR 'ctp17' OR 'cyltezo' OR 'exemptia' OR 'gp 2017' OR 'hulio' OR 'humira' OR 'ibi303' OR 'm 923' OR 'm923' OR 'monoclonal antibody d2e7' OR 'sb 5' OR 'sb5' OR 'amgevita' OR 'amjevita' OR 'adalimumab-bwwd' OR 'adaly' OR 'adalimumab' OR 'infliximab'/exp OR 'inflectra' OR 'infliximab' OR 'remicade' OR 'remsima' OR 'renflexis' OR 'methotrexate'/exp OR '4 amino 10 methylpteroylglutamic acid' OR '4 amino n10 methylpteroylglutamic acid' OR 'mtx' OR 'amethopterin' OR 'amethopterine' OR 'canceren' OR 'farmotrex' OR 'folex' OR 'imeth' OR 'metex' OR 'methotrexat' OR 'methotrexate' OR 'methrotrexate' OR 'methylaminopterin' OR 'meticil' OR 'metothrexate' OR 'metotrexat' OR 'metotrexate' OR 'metrex' OR 'r 9985' OR 'rheumatrex' OR 'texate' OR 'trexall' OR 'xatmep' OR 'xaken' OR 'rituximab'/exp OR 'mabthera' OR 'reditux' OR 'ritemvia' OR 'ritumax' OR 'rituxan' OR 'rituximab' OR 'rituxin' OR 'rituzena' OR 'rixathon' OR 'riximyo' OR 'ruxience' OR 'tuxella' OR 'truxima' OR 'ocrelizumab'/exp OR 'ocrelizumab' OR 'ocrevus' OR 'ofatumumab'/exp OR 'arzerra' OR 'humaxcd20' OR 'ofatumumab' OR 'kesimpta' OR 'ublituximab'/exp OR 'utuxin' OR 'ublituximab' OR 'dmard'		
O	'cost benefit analysis'/exp OR 'cost analysis' OR 'cost benefit' OR 'cost benefit analysis' OR 'cost benefit ratio' OR 'cost-benefit analysis' OR 'cost minimization analysis'/exp OR 'cost minimization' OR 'cost minimization analysis' OR ‘quality of life’ OR ‘QALY’ OR ‘quality adjusted’ OR ‘life year’ OR ‘life years’ OR ‘DALY’ OR ‘disability adjusted’ OR ‘ICER’ OR ‘ICERS’ OR INB OR 'cost effectiveness analysis'/exp OR 'cost effectiveness' OR 'cost effectiveness analysis' OR 'cost effectiveness ratio' OR 'cost efficiency analysis' OR ‘willingness to pay’ OR 'cost utility analysis'/exp OR 'cost utility' OR 'cost utility analysis'	877,755	970,430
PICS	PIO	4,822	5,295
	PIO with #5 AND ('crohn disease'/dm OR 'rheumatic disease'/dm OR 'rheumatoid arthritis'/dm) AND 'human'/de AND ('article'/it OR 'article in press'/it) AND ([adult]/lim OR [aged]/lim OR [middle aged]/lim OR [very elderly]/lim OR [young adult]/lim)	863	1,000
	From 2021 to 5th May 2022		157

PICOS	Scopus search terms	Hits on date 12th Feb 2021	Hits on date 5th May 2022
P	"Rheumatoid arthritis" OR rheumatoid	560,511 results	611,784 results
I	tnf OR "Tumor Necrosis Factor" OR "JAK inhibitor" OR "JAK inhibitors" OR "Janus kinase inhibitor" OR DMARD OR "disease modifying anti-rheumatic drugs" OR biologics OR upadacitinib OR Rinvoq OR baricitinib OR Olumiant OR Simponi OR "Simponi Aria" OR golimumab OR "certolizumab pegol" OR certolizumab OR Inflectra OR infliximab-dyyb OR infliximab OR Remicade OR etanercept-szzs OR Erelzi OR etanercept OR Enbrel OR adalimumab-atto OR Amjevita OR adalimumab OR Humira OR Cyltezo OR Hyrimoz OR Cimzia OR methotrexate OR Amethopterin OR MTX OR Otrexup OR Trexall OR Rheumatrex OR Rasuvo OR tofacitinib OR Xeljanz OR Rituximab OR Rituxan OR Truxima OR Mabthera OR Ocrelizumab OR Ofatumumab OR Ublituximab	1,733,118 results	1,902,804 results
O	"cost effectiv*" OR "cost utility" OR "cost benefit" OR "cost-benefit" OR "quality adjusted life years" OR qaly OR ly OR "life year$" OR daly OR "disability adjusted" OR "incremental cost effective ratio" OR "ICER" OR "incremental net benefit" OR inb OR "benefit ratio" OR 'cost benefit' OR 'cost minimi?ation' OR "cost-effectiveness" OR "cost effectiveness ratio" OR "cost efficiency analys?s"	586,742 results	639,644 results
PIO		6,099 results	
	TITLE-ABS-KEY ("Rheumatoid arthritis" OR rheumatoid) AND (tnf OR "Tumor Necrosis Factor" OR "JAK inhibitor" OR "JAK inhibitors" OR "Janus kinase inhibitor" OR dmard OR "disease modifying anti-rheumatic drugs" OR biologics OR upadacitinib OR rinvoq OR baricitinib OR olumiant OR simponi OR "Simponi Aria" OR golimumab OR "certolizumab pegol" OR certolizumab OR inflectra OR infliximab-dyyb OR infliximab OR remicade OR etanercept-szzs OR erelzi OR etanercept OR enbrel OR adalimumab-atto OR amjevita OR adalimumab OR humira OR cyltezo OR hyrimoz OR cimzia OR methotrexate OR amethopterin OR mtx OR otrexup OR trexall OR rheumatrex OR rasuvo OR tofacitinib OR xeljanz OR rituximab OR rituxan OR truxima OR mabthera OR ocrelizumab OR ofatumumab OR ublituximab) AND ("cost effectiv*" OR "cost utility" OR "cost benefit" OR "cost-benefit" OR "quality adjusted life years" OR qaly OR ly OR "life year$" OR daly OR "disability adjusted" OR "incremental cost effective ratio" OR "ICER" OR "incremental net benefit" OR inb OR "benefit ratio" OR 'cost AND benefit' OR 'cost AND minimi?ation' OR "cost-effectiveness" OR "cost effectiveness ratio" OR "cost efficiency analys?s" OR "cost utility") AND (LIMIT-TO (DOCTYPE , "ar")) AND (LIMIT-TO (SRCTYPE , "j"))	1,542 results	1,716 results
	**From 2021 to 5th May 2022**<br>TITLE-ABS-KEY ("Rheumatoid arthritis" OR rheumatoid) AND (tnf OR "Tumor Necrosis Factor" OR "JAK inhibitor" OR "JAK inhibitors" OR "Janus kinase inhibitor" OR dmard OR "disease modifying anti-rheumatic drugs" OR biologics OR upadacitinib OR rinvoq OR baricitinib OR olumiant OR simponi OR "Simponi Aria" OR golimumab OR "certolizumab pegol" OR certolizumab OR inflectra OR		171 results

	infliximab-dyyb OR infliximab OR remicade OR etanercept-szzs OR erelzi OR etanercept OR enbrel OR adalimumab-atto OR amjevita OR adalimumab OR humira OR cyltezo OR hyrimoz OR cimzia OR methotrexate OR amethopterin OR mtx OR otrexup OR trexall OR rheumatrex OR rasuvo OR tofacitinib OR xeljanz OR rituximab OR rituxan OR truxima OR mabthera OR ocrelizumab OR ofatumumab OR ublituximab) AND ("cost effectiv*" OR "cost utility" OR "cost benefit" OR "cost-benefit" OR "quality adjusted life years" OR qaly OR ly OR "life year$" OR daly OR "disability adjusted" OR "incremental cost effective ratio" OR "ICER" OR "incremental net benefit" OR inb OR "benefit ratio" OR 'cost AND benefit' OR 'cost AND minimi?ation' OR "cost-effectiveness" OR "cost effectiveness ratio" OR "cost efficiency analys?s" OR "cost utility") AND (LIMIT-TO (SRCTYPE , "j")) AND (LIMIT-TO (DOCTYPE , "ar")) AND (LIMIT-TO (PUBYEAR , 2022) OR LIMIT-TO (PUBYEAR , 2021))		

Appendix II: Methods

A) Incremental net benefit (INB) can be estimated as follows:

$$INB = Kx\Delta E - \Delta C \text{ ----------------- } (1)$$

or

$$INB = \Delta E(K - ICER) \text{ ------------------ } (2)$$

$$Var(INB) = K^2\,\sigma_{\Delta E}^2 + \sigma_{ICER}^2 \text{ ------------ } (3)$$

or

$$Var(INB) = K^2\sigma_{\Delta E}^2 + \sigma_{\Delta C}^2 - 2K\rho_{\Delta E\Delta C} \text{ -------- } (4)$$

K is the Willingness to pay (WTP), ΔC and ΔE are incremental cost and incremental effectiveness, $\sigma^2_{\Delta C}$, $\sigma^2_{\Delta E,}$ $\rho_{\Delta C\Delta E}$ were variances of ΔC and ΔE and their covariance, and σ^2_{ICER} was variance of ICER. The WTP was used as reported in the original included studies, i.e., a standard/country specific or GDP based WTP threshold. A positive INB favours treatment, i.e., intervention is cost-effective, whereas a negative INB favours the comparator, i.e., intervention is not cost-effective.

Currency conversions and standardization

The monetary units were converted to purchasing power parity (PPP), adjusted to US$ for the year 2021 before INB calculation. For instance, if a study reported cost, ICER, and thresholds in Euros for 2012, this currency was first converted to 2021 Euros using the historical consumer price index (CPI) of that country. The Euro 2021 value was next converted to PPP adjusted US$ rate using conversion rates from the International Monetary Fund[25]. In addition, the K value from GDP-based threshold was corrected for both latest CPI (2021) and PPP, while for standard/country specific or fixed K, only PPP was corrected. For the variance monetary value conversion, the specific study variance was multiplied by the square of total factors (i.e., CPI and PPP) for the year

2021. For example, if Y is variance of ICER in Euros 2012, this was converted into 2021 PPP adjusted US$ as

$$Var_{PPP_{2021}} = Var_{Euros_{2012}} x \left(\frac{CPI_{Euros_{2021}}}{CPI_{Euros_{2012}}} x \frac{1}{PPP_{2021}} \right)^2 - - - - - - (5)$$

B) Meta-analysis

i. A fixed effect model

$$\mathrm{INB_p} = \frac{\sum_{i=1}^{S} \mathrm{w_i} . \mathrm{INB_i}}{\sum_{i=1}^{S} \mathrm{w_i}} \text{----------------------(1)}$$

$$w_i = \frac{1}{Var(INB_i)} \text{----------------------------(2)}$$

ii. A random effect model

$$\mathrm{INB_p} = \frac{\sum_{i=1}^{S} w_i^* . \mathrm{INB_i}}{\sum_{i=1}^{S} w_i^*} \text{----------------------(3)}$$

$$w_i^* = \frac{1}{Var(INB_i) + \tau^2} \text{-----------------------(4)}$$

$$\tau^2 = \frac{Q - (S-1)}{\sum w_i - \frac{\sum w_i^2}{\sum w_i}} \text{---------------------------(5)}$$

Q is the Cochrane Q-statistic, where Q = 0 if Q < S-1; and s is the number of included studies/comparisons. The heterogeneity of INB was assessed using Cochrane Q-test and I^2 statistic calculated as equations below.

$$\mathrm{Q} = \sum_{i=1}^{S} \mathrm{w_i} \left(\mathrm{INB_i} - \mathrm{INB}_p \right)^2 \text{-------------(6)}$$

$$\mathrm{I}^2 = 100\% \mathrm{x} \, \frac{\mathrm{Q} - (\mathrm{S}-1)}{\mathrm{Q}} \text{----------------------(7)}$$

C) Scenarios developed to obtain variance

- Scenario-1: studies which reports the point estimates & variances for every parameter required for calculation
- Scenario-2: studies which reports the means and 95% CIs of incremental costs & outcomes, and ICER

$$95\%\ CI\ of\ \mu_{ICER} = \hat{\mu}_{ICER} \pm Z_{\alpha/2} \times SE$$

$$UL_{ICER} = \hat{\mu}_{ICER} \pm Z_{\alpha/2} \times SE$$

$$SE = \frac{UL_{ICER} - \hat{\mu}_{ICER}}{Z_{\alpha/2}}$$

$$\hat{\sigma}^2{}_{ICER} = SE^2$$

UL_{ICER} = Upper limit of ICER

$Z_{\alpha/2}$ = Standard Normal = 1.96

$\hat{\mu}_{ICER}$ = mean ICER

- Scenario-3: studies which reports means and 95% CI of costs/outcomes, or ΔC& ΔE, but not ICER or its variance.

  Monte Carlo simulation with a gamma and normal distributions for ΔC and ΔE is performed to estimate covariance between ΔC and ΔE.

- Scenario-4: studies which does not report any dispersion, but provides the CE plane graphs,

  Data can be directly extracted from the CE plane using Web-Plot Digitizer software. The means of ΔC, ΔE, and their variances and co-variance can be estimated accordingly.

- Scenario-5: The study reports only the means (or point estimates) of costs, outcomes, and ICER.

  The measures of dispersions can be borrowed from another similar study if they fulfil the following criteria:

  - They are in the same stratum of country income level, perspective, intervention, comparator, time period, country region, model type, and inputs (i.e., discounting, time horizon).
  - Their ICERs are not much different, e.g., ±50% to 75%

Appendix III: Summary of Findings of GRADE Assessment

Evidence Profile using Grading of Recommendation, Assessment, Development, and Evaluation (GRADE) instrument

P: Adult patients with moderate to severe RA
I: JAK inhibitors alone or combination/sequence with csDMARDs
C: Any others
O: Incremental cost-effectiveness ratio (ICER), or Incremental Net Benefit.

Outcome: Cost-effectiveness (assessed with meta-analysis of cost utility analysis)									
**Quality assessment***						**Summary of findings**			**Comments**
**No of studies**	**Risk of Bias**	**Inconsistency**	**Indirectness**	**Imprecision**	**Publication Bias**	**Effect (US$) INB**	**Effect (US$) 95%CI**	**Certainty/Quality**	
Cost-effectiveness of JAKi compared to csDMARD/bDMARD (Assessed with meta-analysis).									
15	not serious	serious [a]	serious [b]	serious [c]	unlikely	19,886	(1,635 to 38,137)	⊕⊕○○ Very Low	Less evidence from low-middle income countries and high unexplained heterogeneity. Varying population with sequential treatment strategy.
Cost-effectiveness of Second line JAK-i vs csDMARDs/bDMARDs for csDMARD failed RA patients (Assessed with meta-analysis).									
13	not serious	serious [a]	serious [b]	serious [c]	unlikely	23,144	(74.1 to 46,214)	⊕⊕○○ Low	Less evidence from low-middle income countries and high unexplained heterogeneity. Varying population.
Cost-effectiveness of JAKi compared to others from societal perspective (Assessed with meta-analysis).									
4	not serious	not serious	not serious	serious	unlikely	9,976	(6,596 to 13,355)	⊕⊕○○ Low	Less number of studies. Varying population with sequential treatment strategy.
Cost-effectiveness of JAKi compared to others from high income countries (Assessed with meta-analysis).									
10	not serious	serious [a]	serious	serious [c]	unlikely	31,502	(6,440 to 56,564)	⊕⊕○○ Low	High unexplained heterogeneity. Varying population with sequential treatment strategy.
Cost-effectiveness of JAKi compared to others from lifetime horizon (Assessed with meta-analysis).									
14	not serious	serious [a]	serious [b]	serious [c]	unlikely	20,281	(1,855 to 38,707)	⊕⊕○○ Low	Less evidence from low-middle income countries and high unexplained heterogeneity. Varying population with sequential treatment strategy.

[a] high heterogeneity [b] studies included have reported a wide confidence intervals [c] Lack of generalisability

Appendix IV: PRISMA Checklist for Abstract

Section and Topic	Item #	Checklist item	Reported (Yes/No)
**TITLE: Cost-effectiveness of Janus Kinase inhibitors for Rheumatoid Arthritis: A systematic review and meta-analysis of cost-utility studies**			
Title	1	Identify the report as a systematic review.	Yes, Page 1
**BACKGROUND**			
Objectives	2	Provide an explicit statement of the main objective(s) or question(s) the review addresses.	Yes, Page 2
**METHODS**			
Eligibility criteria	3	Specify the inclusion and exclusion criteria for the review.	Yes, Page 2
Information sources	4	Specify the information sources (e.g. databases, registers) used to identify studies and the date when each was last searched.	Yes, Page 2
Risk of bias	5	Specify the methods used to assess risk of bias in the included studies.	Yes, Page 2
Synthesis of results	6	Specify the methods used to present and synthesise results.	Yes, Page 2
**RESULTS**			
Included studies	7	Give the total number of included studies and participants and summarise relevant characteristics of studies.	Yes, Page 2
Synthesis of results	8	Present results for main outcomes, preferably indicating the number of included studies and participants for each. If meta-analysis was done, report the summary estimate and confidence/credible interval. If comparing groups, indicate the direction of the effect (i.e. which group is favoured).	Yes, Page 2
**DISCUSSION**			
Limitations of evidence	9	Provide a brief summary of the limitations of the evidence included in the review (e.g. study risk of bias, inconsistency and imprecision).	Yes, Page 2
Interpretation	10	Provide a general interpretation of the results and important implications.	Yes, Page 2
**OTHER**			
Funding	11	Specify the primary source of funding for the review.	Yes, Page 2
Registration	12	Provide the register name and registration number.	Yes, Page 2

From: Page MJ, McKenzie JE, Bossuyt PM, Boutron I, Hoffmann TC, Mulrow CD, et al. The PRISMA 2020 statement: an updated guideline for reporting systematic reviews. BMJ 2021;372:n71. doi: 10.1136/bmj.n71

Appendix V: PRISMA checklist

Section and Topic	Item #	Checklist item	Location where item is reported
**TITLE: Cost-effectiveness of Janus Kinase inhibitors for Rheumatoid Arthritis: A systematic review and meta-analysis of cost-utility studies**			
Title	1	Identify the report as a systematic review.	Yes, Page 1
**ABSTRACT**			
Abstract	2	See the PRISMA 2020 for Abstracts checklist.	Yes
**INTRODUCTION**			
Rationale	3	Describe the rationale for the review in the context of existing knowledge.	Yes, Page 3
Objectives	4	Provide an explicit statement of the objective(s) or question(s) the review addresses.	Yes, Page 3 to 4
**METHODS**			
Eligibility criteria	5	Specify the inclusion and exclusion criteria for the review and how studies were grouped for the syntheses.	Yes, Page 4
Information sources	6	Specify all databases, registers, websites, organisations, reference lists and other sources searched or consulted to identify studies. Specify the date when each source was last searched or consulted.	Yes, Page 4
Search strategy	7	Present the full search strategies for all databases, registers and websites, including any filters and limits used.	Yes, Page 4 to 5, Appendix 1
Selection process	8	Specify the methods used to decide whether a study met the inclusion criteria of the review, including how many reviewers screened each record and each report retrieved, whether they worked independently, and if applicable, details of automation tools used in the process.	Yes, Page 4 to 5, Figure 1
Data collection process	9	Specify the methods used to collect data from reports, including how many reviewers collected data from each report, whether they worked independently, any processes for obtaining or confirming data from study investigators, and if applicable, details of automation tools used in the process.	Yes, Page 4 to 5
Data items	10a	List and define all outcomes for which data were sought. Specify whether all results that were compatible with each outcome domain in each study were sought (e.g. for all measures, time points, analyses), and if not, the methods used to decide which results to collect.	Yes, Page 5 to 6
	10b	List and define all other variables for which data were sought (e.g. participant and intervention characteristics, funding sources). Describe any assumptions made about any missing or unclear information.	Yes,
Study risk of bias assessment	11	Specify the methods used to assess risk of bias in the included studies, including details of the tool(s) used, how many reviewers assessed each study and whether they worked independently, and if applicable, details of automation tools used in the process.	Yes, Page 6
Effect measures	12	Specify for each outcome the effect measure(s) (e.g. risk ratio, mean difference) used in the synthesis or presentation of results.	Yes, Page 5 to 6, Appendix II
Synthesis methods	13a	Describe the processes used to decide which studies were eligible for each synthesis (e.g. tabulating the study intervention characteristics and comparing against the planned groups for each synthesis (item #5)).	Yes, Page 5 to 6, Appendix II
	13b	Describe any methods required to prepare the data for presentation or synthesis, such as handling of missing summary statistics, or data conversions.	Yes, Page 5 to 6, Appendix II
	13c	Describe any methods used to tabulate or visually display results of individual studies and syntheses.	Yes, Page 5 to 6, Appendix II
	13d	Describe any methods used to synthesize results and provide a rationale for the choice(s). If meta-analysis was performed, describe the model(s), method(s) to identify the presence and extent of statistical heterogeneity, and software package(s) used.	Yes, Page 5 to 6, Appendix II
	13e	Describe any methods used to explore possible causes of heterogeneity among study results (e.g. subgroup analysis, meta-regression).	Yes, Page 5 to 6, Appendix II
	13f	Describe any sensitivity analyses conducted to assess robustness of the synthesized results.	Yes, Page 5 to 6, Appendix II
Reporting bias assessment	14	Describe any methods used to assess risk of bias due to missing results in a synthesis (arising from reporting biases).	Yes, Page 6
Certainty assessment	15	Describe any methods used to assess certainty (or confidence) in the body of evidence for an outcome.	Yes, Page 6

Section and Topic	Item #	Checklist item	Location where item is reported
**RESULTS**			
Study selection	16a	Describe the results of the search and selection process, from the number of records identified in the search to the number of studies included in the review, ideally using a flow diagram.	Yes, Page 6, Figure 1
	16b	Cite studies that might appear to meet the inclusion criteria, but which were excluded, and explain why they were excluded.	Yes, Page 6, 7
Study characteristics	17	Cite each included study and present its characteristics.	Yes, Page 6, 7 & Table 1
Risk of bias in studies	18	Present assessments of risk of bias for each included study.	Yes, Page 8 & Supp Fig 1
Results of individual studies	19	For all outcomes, present, for each study: (a) summary statistics for each group (where appropriate) and (b) an effect estimate and its precision (e.g. confidence/credible interval), ideally using structured tables or plots.	Yes, Page 6 to 7
Results of syntheses	20a	For each synthesis, briefly summarise the characteristics and risk of bias among contributing studies.	Yes, Page 8
	20b	Present results of all statistical syntheses conducted. If meta-analysis was done, present for each the summary estimate and its precision (e.g. confidence/credible interval) and measures of statistical heterogeneity. If comparing groups, describe the direction of the effect.	Yes, Page 8 to 10 & Figure 2,3 & Supp Fig 5 to 13
	20c	Present results of all investigations of possible causes of heterogeneity among study results.	Yes, Page 8 & Supp Fig 4 to 10
	20d	Present results of all sensitivity analyses conducted to assess the robustness of the synthesized results.	Yes, Page 8 & Supp Fig 4 to 13
Reporting biases	21	Present assessments of risk of bias due to missing results (arising from reporting biases) for each synthesis assessed.	Yes, Page 8 & Supp Fig 1
Certainty of evidence	22	Present assessments of certainty (or confidence) in the body of evidence for each outcome assessed.	Yes, Page 10, & Appendix III
**DISCUSSION**			
Discussion	23a	Provide a general interpretation of the results in the context of other evidence.	Yes, Page 11
	23b	Discuss any limitations of the evidence included in the review.	Yes, Page 12
	23c	Discuss any limitations of the review processes used.	Yes, Page 12
	23d	Discuss implications of the results for practice, policy, and future research.	Yes, Page 12,13
**OTHER INFORMATION**			
Registration and protocol	24a	Provide registration information for the review, including register name and registration number, or state that the review was not registered.	Yes, Page 4
	24b	Indicate where the review protocol can be accessed, or state that a protocol was not prepared.	Yes, Page 4
	24c	Describe and explain any amendments to information provided at registration or in the protocol.	NA
Support	25	Describe sources of financial or non-financial support for the review, and the role of the funders or sponsors in the review.	Yes, Page 1
Competing interests	26	Declare any competing interests of review authors.	Yes, Page 1
Availability of data, code and other materials	27	Report which of the following are publicly available and where they can be found: template data collection forms; data extracted from included studies; data used for all analyses; analytic code; any other materials used in the review.	NA

From: Page MJ, McKenzie JE, Bossuyt PM, Boutron I, Hoffmann TC, Mulrow CD, et al. The PRISMA 2020 statement: an updated guideline for reporting systematic reviews. BMJ 2021;372:n71. doi: 10.1136/bmj.n71
